# Electroacupuncture alleviates perioperative hypothalamus-pituitary-adrenal axis dysfunction *via* circRNA-miRNA-mRNA networks

**DOI:** 10.3389/fnmol.2023.1115569

**Published:** 2023-01-25

**Authors:** Yu Wang, Wei Hu, Jing Han, Jiayuan Zheng, Ning Jiang, Yi Feng, Zhanzhuang Tian

**Affiliations:** ^1^Department of Integrative Medicine and Neurobiology, School of Basic Medical Sciences, Shanghai Key Laboratory for Acupuncture Mechanism and Acupoint Function, State Key Laboratory of Medical Neurobiology, Institute of Acupuncture and Moxibustion, Fudan University, Shanghai, China; ^2^State Key Laboratory of Genetic Engineering, School of Life Science, Fudan University, Shanghai, China

**Keywords:** hypothalamic–pituitary–adrenal axis, electroacupuncture, surgical trauma, RNA sequencing, circRNA-miRNA-mRNA network

## Abstract

Electroacupuncture (EA) has long been used to alleviate surgery-induced hypothalamic–pituitary–adrenal axis dysfunction. However, its downstream gene targets in the brain remain unclear. The aim of the present study was to clarify the potential targets of EA based on RNA sequencing techniques (RNA-seq). Rats were divided into normal control (NC), hepatectomy surgery (HT), HT + EA, and HT + sham EA groups followed by RNA-seq of two representative nuclei in the hypothalamus and amygdala. Weighted Gene Co-expression Network Analysis and Gene Set Enrichment Analysis identified six gene modules associated with neuroendocrine transmitters and neural remodeling in the hypothalamus. Furthermore, circRNA-miRNA-mRNA interaction networks revealed EA-related candidate miRNAs and circRNAs, of which opioid receptor mu 1 might be an EA-specific target, and showed regulation by competing endogenous RNA. We identified the neuroendocrine circRNA-miRNA-mRNA networks through which EA has an effect on HPA axis dysfunction, thus providing potential targets and future research directions for EA treatment.

## 1. Introduction

The hypothalamic–pituitary–adrenal (HPA) axis plays an important role in stress responses (Heck and Handa, 2019; Frankiensztajn et al., 2020), and HPA hyperactivity is associated with surgery-induced acute stress in clinical practice (Spencer-Segal et al., 2020; Zhu et al., 2021). Previous studies have shown that surgical trauma aggravates hyperactivity of the HPA axis, which is characterized by excessive release of hypothalamic corticotrophin-releasing hormone (CRH), peripheral adrenocorticotropic hormone (ACTH), and corticosterone (CORT; Qin et al., 2021). The pathophysiological events associated with the HPA axis can also trigger secondary effects such as abnormal metabolism (Mi et al., 2019), immunosuppression (Kim et al., 2019), neuroinflammation (Cernackova et al., 2020), hypertension (Hepsen et al., 2020), and multiple system dysfunction (Keller et al., 2017; Juruena et al., 2018). As a vital neuroendocrine nucleus of the hypothalamus, the periventricular nucleus (PVN) is widely distributed with CRH neurons and functions as the initial part of the HPA axis (Daviu et al., 2020). In addition to the PVN, the central nucleus of the amygdala (CEA) acts as the physiological response center and also plays a major role in stress mainly through changes in GABAergic neurons (Sharp, 2017). There are nerve fibers projecting to the PVN from the CEA, and these can activate neurons in the PVN (Beal et al., 1985). In stress-related situations, the CEA may assist the PVN in producing “fight-or-flight” responses. Analysis at the transcriptomic level of the hypothalamus as well as its connectivity with other brain regions like the amygdala also remain unclear and are significance topics for further research.

Electroacupuncture (EA) has long been used to treat HPA axis dysfunction, potentially through the regulation of hormones and inflammatory factors (Han et al., 2021; Zhou et al., 2022). Clinical trials have shown that EA could relieve preoperative tension and anxiety effectively, thus reducing the use of anesthetics during surgery (Yuan and Wang, 2019). Previous studies also found that EA can be beneficial for treating postoperative pain, stress, and inflammation (Li et al., 2021, 2022; Yang et al., 2021). The mechanisms of EA treatment have been shown to be partly associated with the suppression of hypothalamic CRH secretion together with the inhibitory role of gamma-aminobutyric acid (GABA) receptor subunits, N-methyl-D-aspartate (NMDA) receptor, etc. (Mody and Maguire, 2011; Le et al., 2016; Zhu et al., 2017, 2018; Jiang et al., 2018). Due to the limitations of traditional research methods that focus on single target molecules, the broad changes in gene expression profiles related to EA amelioration of HPA axis hyperactivity have remained poorly understood.

High-throughput sequencing has been used to identify the roles of mRNA and competing endogenous RNA in the regulation of neuroendocrine function. Competing endogenous RNA, especially microRNAs (miRNAs) and circular RNAs (circRNAs), have been found to be widely associated with stress (Du et al., 2019), neuronal development (Ma et al., 2019), and endocrine-related diseases (Deng et al., 2021). In order to explore the potential regulatory targets of EA, a hepatectomy rat model was treated with EA at the Zusanli (ST 36) and Sanyinjiao (SP 6) acupoints. Analytic algorithms including Weighted Gene Co-expression Network Analysis (WGCNA) and Gene Set Enrichment Analysis (GSEA) were also used to explore the sequencing results. Taken together, our study aimed to identify novel transcriptomic targets of EA in the central neural system, which may help to reveal the mechanisms underlying EA treatment and promote its clinical usage.

## 2. Materials and methods

### 2.1. Animals and ethics

Male Sprague–Dawley rats (SPF, 7 weeks of age, 200 ± 20 g) were purchased from the Slack Laboratory Animal Center (Shanghai Branch of the Chinese Academy of Sciences, Shanghai, China) and were kept at a constant temperature of 22–24°C with 12/12 h standard light/dark circadian rhythm and free access to food and water (2–3 rats/cage). All animals were adapted to the experimental environment for 1 week prior to the experiment. All experiments respected the National Institutes of Health Laboratory Animal Care and Use Guidelines (NIH Publication No. 23–80, revised 1996) and were approved by the Ethics Committee of Fudan University in China (20190221–068). All efforts were made to minimize the suffering of the animals.

### 2.2. Modeling and EA treatment

Twelve rats were randomly divided into four groups: normal control (NC), hepatectomy (HT), HT + EA, and HT + sham EA (SEA). For the hepatectomy model preparation, rats were anesthetized with isoflurane (0.5–0.7 L/min), then partial hepatectomy was performed in the HT, HT + EA, and HT + SEA groups. In brief, an incision was made along the midline of the abdomen, the ligaments around the liver were separated, and about 20% of the left liver lobe was removed. For the HT + EA group, EA was performed 24 h before and immediately after the operation at the Zusanli (ST 36, 10 mm above the hind limb medial malleolus front of the tibia and fibula; Lou et al., 2020) and Sanyinjiao (SP 6, 5 mm below the head of the fibula under the knee joint and 2 mm lateral to the anterior tubercle of the tibia) acupoints. Sterilized stainless steel needles (0.22 mm in diameter and 13 mm in length; Hua Tuo, Suzhou, China) were unilaterally inserted into ST 36 and SP 6 to a depth of 3 mm beneath the skin. Then the needles were carefully connected to a HANS Acupoint Nerve Stimulator (LH202H, Beijing, China), and electrical current stimulation (2 Hz for 1.05 s and 15 Hz 2.85 s; 2–3 mA intensity) was applied for 30 min. For the HT + SEA group, needles were unilaterally inserted into ST 36 and SP 6, but without connection to the HANS Acupoint Nerve Stimulator. Animals in each group were habituated to the restraint device (30 min each time) once a day for three consecutive days prior to the surgery. All of the rats were fixed in the restraint device and kept awake during the EA and SEA treatment in order to minimize the side effects of the anesthetic. At the same time, rats in the NC and HT groups were also fixed in the restraint belt for the same time as the rats in the HT + EA and HT + SEA groups. After sacrifice, the PVN of the hypothalamus and the CEA were extracted based on the Allen Atlas. The PVN is located in the ventral surface of the brain, below the thalamus, on both sides of the third ventricle, while the CEA is an oval gray matter nucleus complex located in the temporal lobe of the brain anterior to the uncinate gyrus. All samples for molecular biology experiments such as Quantitative real-time PCR (qRT-PCR), western blot (WB), enzyme linked immunosorbent assay (ELISA), and sequencing were taken 24 h after the last EA treatment. Due to the limited samples provided by one rat, the brain samples were not from the same animal, but the same batch. Brains were collected at the same time to ensure the homogeneity of the samples.

### 2.3. qRT-PCR

The total RNA of the PVN and CEA was extracted with TRIzol reagent (15,596,018, Thermo Fisher Scientific, Carlsbad, CA, United States), and 1 μg of hypothalamic total RNA was reverse-transcribed into cDNA (RR036A, PrimeScript RT Master Mix, TaKaRa, Japan). qRT-PCR amplification with the TB Green Kit (RR420A, TB Green Premix Ex Taq, TaKaRa, Japan) was performed. The primers for CRH and GAPDH (Supplementary Table 1) were synthesized by Shanghai Sangon Biological Engineering Technology Company (Shanghai, China) and gene expression was measured with an Applied Biosystem 7,500 Real-Time PCR system. GAPDH was used as the internal control and data were analyzed by the 2^–△△Ct^ method.

### 2.4. WB

The PVN tissue was lysed with RIPA buffer (P0013B, Beyotime, China) containing PMSF (ST506, Beyotime, China) on ice for 30 min. The protein samples were loaded onto 12% SDS-PAGE gels, electrophoresed, and transferred to PVDF membranes (Millipore Sigma, Burlington, MA, United States). The PVDF membranes were blocked with 5% (w/v) nonfat milk at room temperature for 2 h and then incubated overnight at 4°C with primary antibodies against CRH (ab184238, Abcam, Cambridge, United Kingdom) or β-tubulin (10094-1-AP, Proteintech, United States). The PVDF membrane was visualized by enhanced chemiluminescence (ECL kit, WBKLS0500, Millipore, Germany), and the protein concentration was quantified with the Quantity One software (version 4.0.3) from Bio-Rad (Hercules, CA, USA). After the lanes on the image were defined, the band densities of CRH and β-tubulin were measured separately. The density of the CRH protein band was normalized to the corresponding β-tubulin for each sample.

### 2.5. ELISA

Rats were anesthetized with pentobarbital sodium (40 mg/kg) 24 h after the last EA treatment. Peripheral blood samples were collected through cardiac puncture after ensuring that the rats were under deep anesthesia, as evidenced by lack of response to a painful stimulus, such as toe or tail pinch (Parasuraman et al., 2010). The sternum was carefully cut to fully expose the rat heart after disinfection. About 3 ml of blood was taken from the left ventricle slowly by blood collection needles and tubes. The peripheral blood samples were then centrifuged at 1500 × *g* for 25 min after being placed at room temperature for 1 h. The upper serum was collected after centrifugation and then stored in the −80°C refrigerator until serum hormones were measured. Serum levels of ACTH (BPE30596, Lengton Biological Technology, China) and CORT (BPE30590, Lengton Biological Technology, China) were measured using ELISA kits according to the manufacturer’s instructions. Serum samples and standards were loaded onto 96-well plates in triplicate for each essay, and the absorbance was measured at 450 nm.

### 2.6. Transcriptome sequencing and data pre-analysis

Total RNA was extracted from tissue samples of the PVN and CEA, and the preparation of the cDNA library and the sequencing were performed by Novo Gene Biotech Co. Ltd. (Beijing, China). A strand-specific library strategy was used to construct the mRNA and circRNA libraries. Sequencing data for the miRNA library were generated using a NEBNext Multiplex Small RNA Library Prep Set for Illumina® (NEB, United States). The insert size of the library was examined using an Agilent 2100 bioanalyzer (Agilent Technologies, Santa Clara, CA, United States), and only effective concentrations >2 nM were used for sequencing. The brain samples yielded 24 RNA libraries, and an illumina Hiseq4000/Hiseq X Ten (Illumina, San Diego, CA, United States) was used following the principle of sequencing by synthesis. The raw reads were filtered to obtain the clean reads after removing adapter-related reads, reads containing uncertain bases, and low-quality reads with base quality phred score ≤ 20. The clean reads were then mapped to the rat reference genome (mRatBN7.2) using HISAT2 algorithms and were quantitatively analyzed using the featureCounts software. Around 90% of the total mapped reads were assigned to the annotated genes. Differential expression analysis was performed with DESeq2, and the threshold of significance was set as a *p* value < 0.05. Because HT and EA were assumed to have opposite impacts on gene expression, we further extracted node mRNA (nmRNA) from differentially expressed mRNA (DEmRNA) by taking the intersection between HT and EA with opposite expression trends.

### 2.7. Principle component analysis and functional enrichment analysis

Principle component analysis (PCA) was conducted to assess the differences among groups and the duplication within groups based on the FPKM value using the prcomp package in R. Functional enrichment analysis including Kyoto Encyclopedia of Genes and Genomes (KEGG) pathways and Gene Ontology (GO) terms were performed using the clusterProfiler package in which the GO terms were further clustered.

### 2.8. Protein–protein interaction network construction and hub gene selection

A Protein–protein interaction (PPI) network was created based on the nmRNA using the Search Tool for the Retrieval of Interacting Genes (STRING) database (Karimizadeh et al., 2019). In order to visualize the PPI network and identify the hub gene, we used Cytoscape (v3.7.2) and the cytoHubba app, allowing 12 algorithms at the same time to evaluate the most important nodes.

### 2.9. WGCNA and GSEA

A gene co-expression network was constructed based on the whole 24 libraries (one set in the CEA EA group was removed due to very large homogeneous differences), and the soft-thresholding power was set to 8 using an R2 cut-off of 0.8. The 12 clustered modules were then assigned colors, and the known stress-related genes were all put into the correlation analysis, which produced six strongly correlated modules (M1–M6) thus enabling the subgrouping of nmRNA.

GSEA software (v4.2.3) was used for functional enrichment analysis (KEGG and GO) of the six modules following the official guide. In addition, C3 collections from the Molecular Signatures Database v7.5.1 were used for the prediction of miRNA-regulated targets.

### 2.10. Differentially expressed miRNA and circRNA

Differential expression analysis of miRNA and circRNA followed the basic DESeq2 process with a threshold of *p* < 0.05. Using the 348 backtracking miRNAs and 140 DEmiRNAs of the PVN, 35 node miRNAs (nmiRNAs) were further screened out. Using the combination of miRanda, PITA, and RNAhybrid to predict mRNA regulated by the 35 nmiRNAs gave a total of 5,816 mRNAs, among which 444 were DEmRNAs with their GO and KEGG results representing functional enrichment of nmiRNA. Moreover, considering the opposite trends between miRNA and its targeted mRNA, only 164 mRNAs were filtered out and an nmRNA-nmiRNA interaction network was constructed. Similarly, because circRNA theoretically follows the same trends compared with mRNA and the opposite trends compared with miRNA, 189 circRNA-miRNA-mRNA correspondence pathways were further explored. Novel circRNA sequences were aligned with circAtlas 2.0, and 96% of the tested novel circRNA had over 99% similarity compared with the database.

### 2.11. Statistical analysis

GraphPad Prism 7 software (GraphPad Software Inc., San Diego, CA, United States) was used for the statistical analysis. One-way analysis of variance (ANOVA) followed by Tukey’s test or Dunnett’s multiple comparisons test was used to compare the differences between groups. *p* < 0.05 was considered statistically significant for all analyses. All results are presented as the mean ± standard error of the mean (SEM).

## 3. Results

### 3.1. EA alleviated surgical trauma-induced hyperactivity of the HPA axis by affecting gene expression in the CEA and PVN

**Figure 1:**
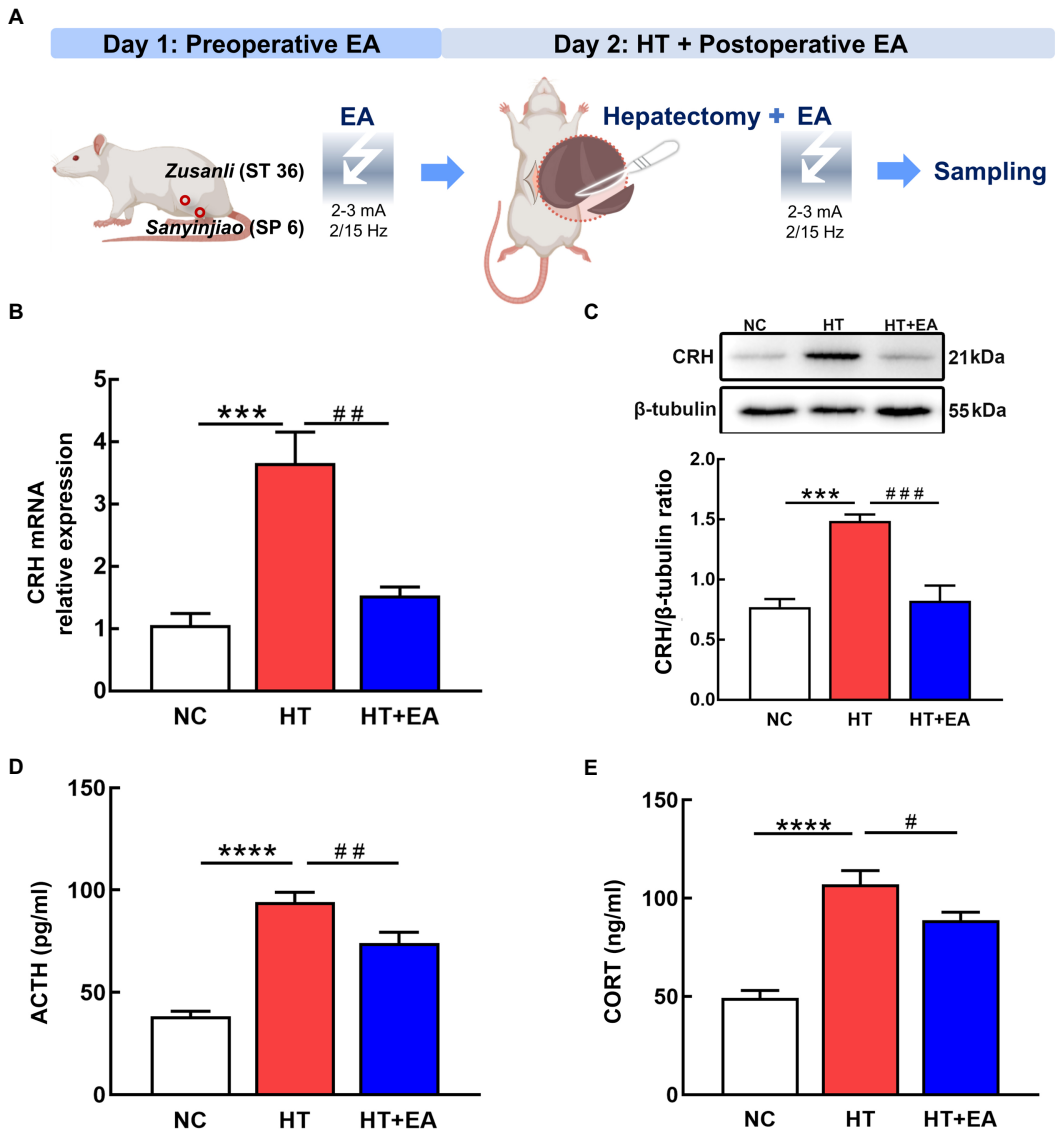
EA relieved the surgery-induced hyperactivity of the HPA axis. **(A)** Flow chart of the HT-induced stress rat model and EA treatment. **(B,C)** Quantification of CRH mRNA and protein detected by RT-PCR and western blot. **(D,E)** Serum levels of ACTH and CORT as measured by ELISA. Data are shown as the mean ± SEM. ^*^p < 0.05, ^**^p < 0.01, ^***^p < 0.001, ^****^p < 0.0001, versus the NC group. ^#^*p* < 0.05, ^##^*p* < 0.01, ^###^*p* < 0.001, and ^####^*p* < 0.0001 versus the HT group.

To clarify the treatment effect of EA on surgical trauma, we first constructed the HT + EA rat model in which EA treatment was performed pre-operatively and post-operatively to relieve surgery-induced HPA axis dysfunction (Figure 1A). The effectiveness of EA was preliminarily evaluated by detecting HPA-related markers, including CRH, ACTH, and CORT. We found that both CRH mRNA and protein in the PVN increased in the HT group but were significantly decreased in the HT + EA group (Figures 1B,C). In addition, there was a similar tendency between the changes of serum hormones (ACTH, CORT) and hypothalamic CRH (Figures 1D,E). Taken together, these results indicated that surgery caused hyperactivity of the HPA axis and that the dysfunction could be relieved by EA.

**Figure 2:**
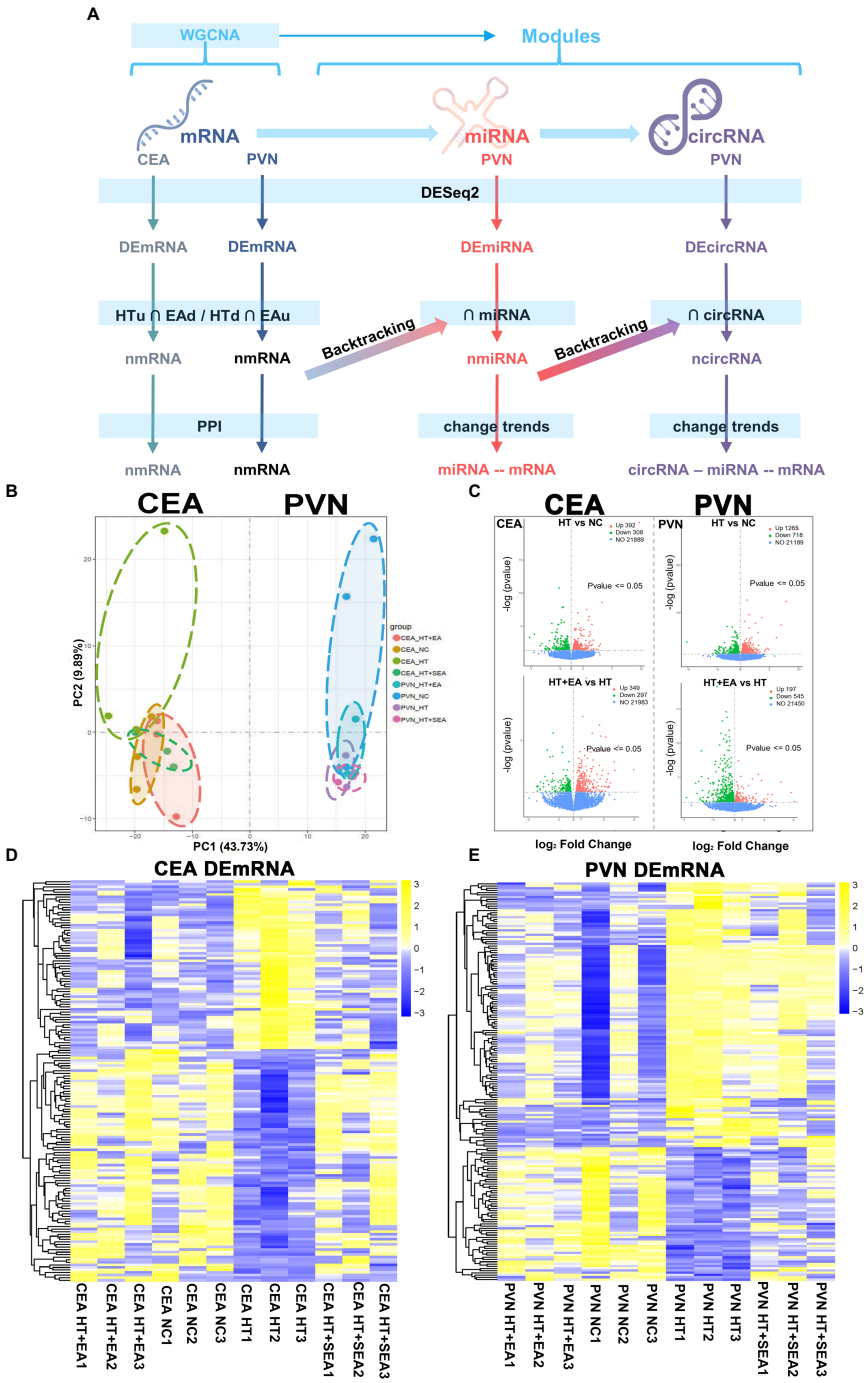
EA at Zusanli (ST36) and Sanyinjiao (SP6) altered the gene expression patterns in the CEA and PVN. **(A)** Flow chart of the analytical process from raw RNAseq data. **(B)** PCA of the CEA and PVN. **(C)** Volcano plots of transcribed genes with a *p* value < 0.05 and |log_2_ (Fold Change) | > 0. **(D,E)** Cluster heatmap of DEmRNAs in the CEA and PVN. Data is available from NCBI GEO: GSE214276, GSE214277, GSE214278, and GSE214279.

RNAseq was used to investigate changes in gene expression profiles in the central nervous system after HT and EA treatment (Figure 2A). The two crucial neuroendocrine nuclei, CEA and PVN, were used to construct the circRNA-miRNA-mRNA interaction network and for the further network analysis. The CEA and PVN were clearly distinguished in the PC1 dimension of the PCA, and different subgroups including NC, HT, HT + EA, and HT + SEA were also independently clustered (Figure 2B). The DEmRNAs were further screened by DESeq2 and shown in volcano plots (Figure 2C). A total of 1,246 DEmRNAs were identified in the CEA, with 392 up-regulated (HTu) and 308 down-regulated (HTd) comparing the HT and NC group, and 349 up-regulated (EAu) and 297 down-regulated (EAd) comparing the HT + EA and HT group (Supplementary Sheet 1). A total of 2,725 DEmRNAs were identified in the PVN, with 1,265 HTu, 718 HTd, 197 EAu, and 545 EAd (Supplementary Sheet 1). A hierarchical clustering heatmap showed a similar gene expression pattern between the NC and HT + EA groups, while obvious differences were observed comparing HT with either the NC or HT + EA groups (Figures 2D,E). Taken together, it appeared that the effect of EA on alleviating surgery-induced HPA axis hyperactivity was correlated to the gene expression profiles in the CEA and PVN.

### 3.2. Identification of EA-related nmRNA in the CEA and PVN

**Figure 3:**
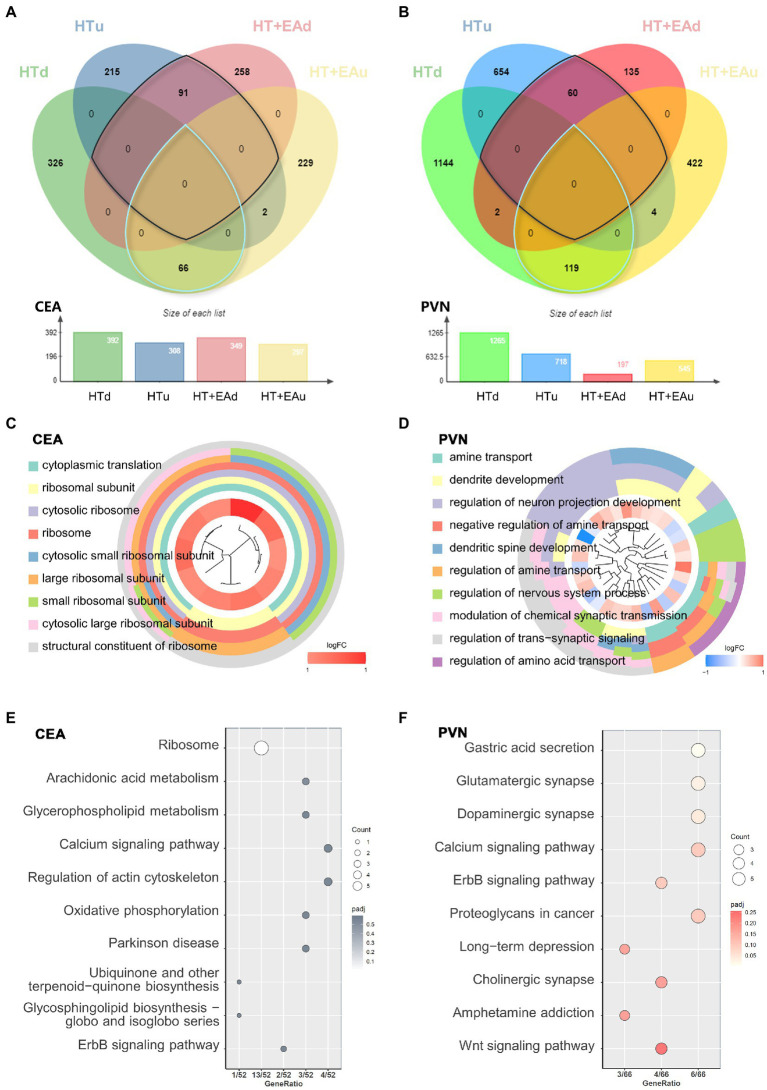
Transcriptional patterns and functional enrichment analysis. **(A,B)** Venn diagrams showing the overlap of DEmRNAs among the HTu, HTd, EAu, and EAd groups. **(C,D)** GO cluster and **(E,F)** KEGG pathway analysis of DEmRNAs in the CEA and PVN.

To further investigate the potential targets of EA in ameliorating HPA axis hyperactivity, we filtered differentially expressed mRNA with opposite trends after HT and EA treatments (Figures 3A,B). The nmRNAs were defined as genes that had opposite expression changes after HT and HT + EA. A total of 157 nmRNAs were identified in the CEA and 179 in the PVN (Supplementary Sheet 2).

GO and KEGG functional enrichment analyses were carried out to identify the potential functions of nmRNAs in both the CEA and PVN. The results of GO clustering showed that nmRNAs in the CEA were mainly related to ribosomal activities such as the cytosolic ribosome and the large ribosomal subunit (Figure 3C). The common biological functions of nmRNAs in the PVN were mostly related to neuron activities such as the regulation of neuron projection development and the modulation of chemical synaptic transmission (Figure 3D). KEGG analysis in the CEA pointed to pathways represented by the ribosome, arachidonic acid, glycerophospholipid metabolism, calcium signaling pathways, etc. (Figure 3E), while KEGG analysis in the PVN showed associations with glutamatergic synapse, dopaminergic synapse, and the calcium and ErbB signaling pathways (Figure 3F). Notably, calcium and ErbB signaling pathways were two overlapping pathways between the CEA and PVN. Other predominant distinctive pathways indicated by either GO or KEGG analysis suggest that EA has different effects in the CEA and PVN to ameliorate HPA axis dysfunction after surgical trauma (Supplementary Sheet 2). In conclusion, the underlying mechanisms through which EA treatment alleviates surgical trauma-induced neuroendocrine disorders may be through changes in ribosomal activity and protein translation in the CEA and changes in neurotransmitter and synapse function in the PVN.

### 3.3. WGCNA distinguished six EA-related modules in the PVN

**Figure 4:**
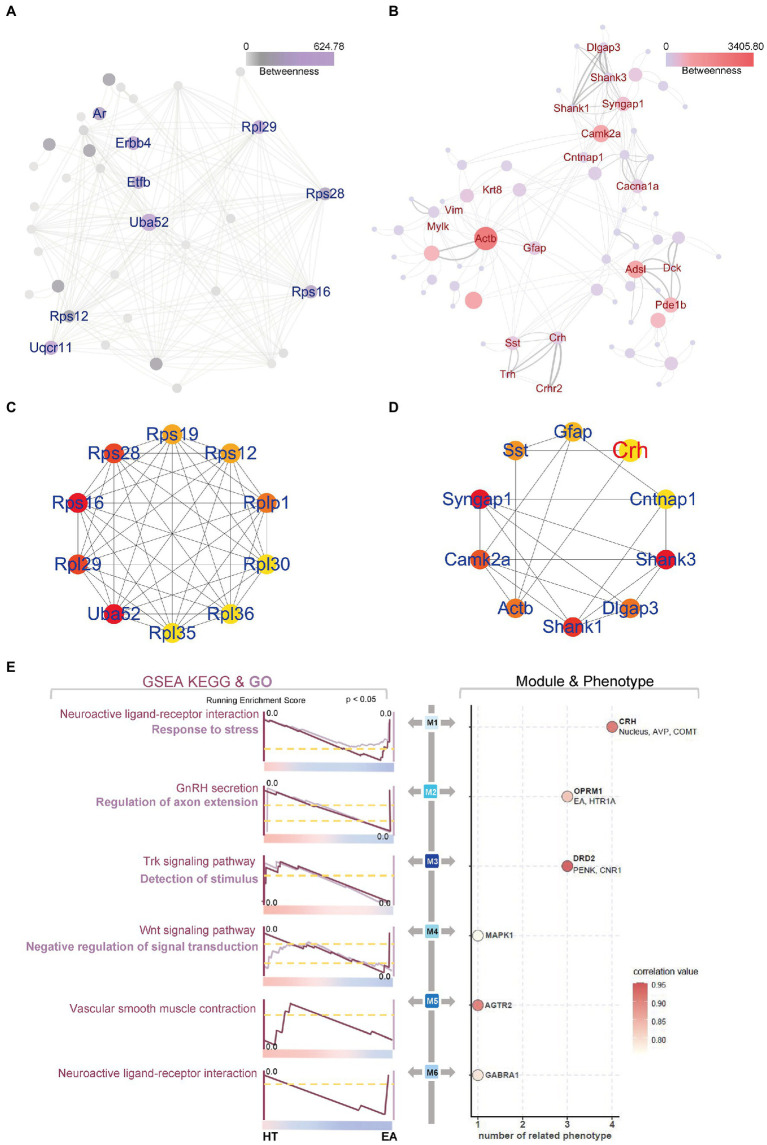
PPI networks and WGCNA further explored EA’s effect on the PVN. **(A,B)** PPI and **(C,D)** Hub genes of DEGs in the CEA and PVN were identified using online STRING tools and the Cytoscape App *CytoHubba*. **(E)** WGCNA and GSEA identified six different modules with specific functions. The top enriched GSEA terms are shown, and the color bar indicates the correlation value. The line thickness and circle size in **(B)** indicated the combined score and betweenness auto-calculated by the Cytoscape software, representing the connectivity between two nodes.

We constructed PPI networks to better visualize the details of the functional nmRNAs involved in the EA treatment. In the CEA, mainly ribosomal proteins made up the hub genes and network proteins (Figures 4A,C). In the PVN, the networks showed more complexity and were clearly clustered into several subgroups (Figures 4B,D). First, *CRH* interacted with *somatostatin*, *Crhr2*, and thyrotropin-releasing hormone (*TRH*) to form an HPA axis and neuroendocrine-related sub-network. Second, synapse remodeling and growth-related genes formed another sub-network, including calcium-dependent protein kinase II alpha (*Camk2a*), *β-actin*, and synaptic Ras GTPase activating protein 1 (*SYNGAP1*). In addition, ErbB signaling in the CEA was characterized by *Erbb4* and calcium signaling in the PVN was characterized by *Camk2a* in the PPI network. Therefore, compared to the changes in ribosome activity and translation in the CEA, EA mainly affected *CRH* as an indicator of neuroendocrine and synapses remodeling in the PVN, indicating that the PVN may play a more important role than the CEA in mediating the effects of EA on alleviating neuroendocrine dysfunction.

To further assess the distinct gene expression patterns in the PVN and CEA, WGCNA was applied using 23 sequencing databases for all groups (Figure 4E). Six essential modules were clustered with close relationships to surgical trauma, nucleus location, or EA treatment. As a result, several neuroendocrine markers stood for M1 to M6, including *CRH*, opioid receptor mu 1 (*OPRM1*), dopamine receptor D2 (*D2R*), mitogen-activated protein kinase 1 (*MAPK1*), angiotensin II receptor type 2 (*AGTR2*), and gamma-aminobutyric acid type A receptor alpha1 subunit (*GABRA1*), respectively. Four modules were related to the positive neurotransmitter regulators, while M6 was related to negative neurotransmitter regulation and M4 was related to the MAPK signaling pathway. Moreover, M1 and M2 also correlated with other factors, indicating the multifactorial synergistic effect, such as arginine vasopressin (*AVP*) and catechol-O-methyltransferase (*COMT*), which were co-expressed with *CRH* in M1, and hydroxytryptamine receptor 1A (*HTR1A*), which was co-expressed with *OPRM1* and was most strongly related to EA in M2.

Moreover, GSEA was used to perform the functional enrichment of M1–M6 based on curated gene set 2 (including KEGG analysis) and curated gene set 5 (including GO analysis) of the Molecular Signatures Database (v7.5.1). The weighted functional enrichment calculated by GSEA supplemented each module with specific potential biological functions (Figure 4E). It was obvious that EA could down-regulate the stress response induced by surgical trauma in each module because all of the peaks for the enrichment scores appeared close to HT. Several types of regulation patterns were revealed in the different modules. First, EA could act through neuroactive ligand-receptor interactions, as represented by positive *CRH* in M1 and negative *GABARA1* in M6. Second, certain signaling pathways were involved in the regulation of neurotransmitters, including *Trk* signaling in M3 and *Wnt* signaling in M4. Third, M2 seemed to correlate with the secretion of gonadotropin-releasing hormone (*GnRH*) in addition to *OPRM1* and *HTR1A*. Lastly, M5 was mainly related to neurovascular regulation through *AGTR2*. Thus, the six EA-related modules were clustered to better explain the multifaceted influence of EA on surgical trauma.

### 3.4. Construction of miRNA-mRNA networks in the PVN

**Figure 5:**
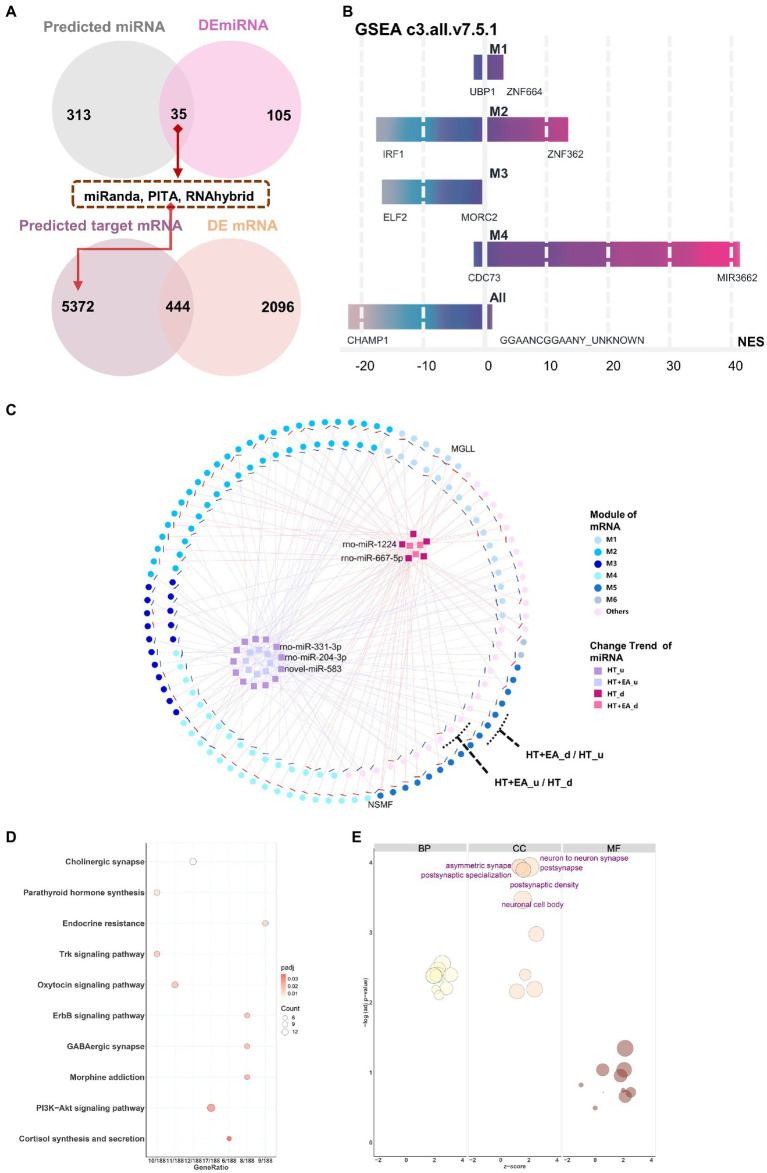
Interaction networks of nmiRNAs and functional enrichment analysis. **(A)** The data flow shows the overlaps for filtering out the nmiRNAs and the targeted genes. **(B)** Potential gene target prediction for six modules using the GSEA curated gene set C3 database (Molecular Signatures Database v7.5.1). M5 and M6 were omitted because of the small number of genes in the sets. **(C)** Interaction networks showed miRNAs with different change trends distributed among the six gene modules, including HT up-or down-regulated (HTu and HTd) and EA up-or down-regulated (EAu, EAd). The top 5 weighted miRNAs were labelled based on their degree of connection with mRNA. **(D,E)** KEGG and GO functional enrichment analysis of miRNA-targeted mRNAs.

Based on the findings of EA-related nmRNAs and modules in the PVN, the regulation of target mRNA by miRNA was further explored. Using miRNA target gene prediction software (miRanda, PITA, and RNA hybrid), 348 miRNAs were predicted from 179 nmRNAs screened in the first stage (Supplementary Sheet 3), followed by taking the intersection with 140 DEmiRNAs. The 35 intersected miRNAs, referred to as nmiRNAs, were selected for further investigation. Similarly, targeted mRNAs were predicted based on the 35 nmiRNAs, followed by taking the intersection with DEmRNAs, which ultimately resulted in 444 mRNAs (Figure 5A).

Functional enrichment analysis and interaction networks were used to explore the function of the 35 nmiRNAs. Curated gene set 3 (regulatory target gene sets) of the GSEA was initially used to detect potential targets of transcription factors or miRNAs for each module. Using the threshold of FDR < 25% and value of *p* < 0.05, only modules M1–M4 were enriched for gene sets (Figure 5B). The most significantly enriched results in both M1 and M2 were related to zinc finger protein-targeted genes, which meant that there was co-regulation of miRNA on the common targeted mRNA binding sites between surgery-induced stress and HT + EA treatment. Furthermore, miRNA-mRNA interaction networks were constructed to better visualize the complex relationships among nmiRNAs, mRNAs, and each module (Figure 5C).

Comparing data collected after HT and HT + EA treatment, the mRNAs with opposite trends were considered to be closely clustered as two outer rings. Two mRNAs – monoglyceride lipase (*MGLL*) and NMDA receptor synaptonuclear signaling and neuronal migration factor (*NSMF*) – were outstanding due to the opposing effects of EA and HT. *MGLL* was up-regulated by rno-miR-3,548 after HT and down-regulated by rno-miR-92b-3p after EA, thus showing involvement of the endocannabinoid signaling pathway to relieve pain. Similarly, *NSMF* was up-regulated by rno-miR-3,099, rno-miR-667-5p, and rno-miR-1,224 after HT and down-regulated by rno-miR-328a-3p after EA, which was related to NMDA-sensitive glutamate receptor signaling and long-lasting remodeling of neural dendrites and spines. The 35 nmiRNAs composing the inner two clusters contained the 5 labeled top-weighted miRNAs, including miR-1224, miR-667-5p, miR-331-3p, miR-204-3p, and novel miR-583. In addition, M2 had the greatest proportion of genes regulated by nmiRNAs, indicating the considerable importance of miRNA regulation of mRNA in response to EA treatment during surgery. In addition to the interaction networks, KEGG and GO analyses were further performed to characterize the function of the 35 nmiRNA-targeted mRNAs. The top enriched KEGG pathway pointed to more specific types of synapses, such as cholinergic, oxytocin, GABAergic, and morphine, together with the ErbB, Trk, and PI3K-Akt signaling pathways (Figure 5D). The top enriched GO terms were mainly related to synapse remodeling and postsynaptic function (Figure 5E).

### 3.5. circRNA-miRNA-mRNA networks in the PVN

**Figure 6:**
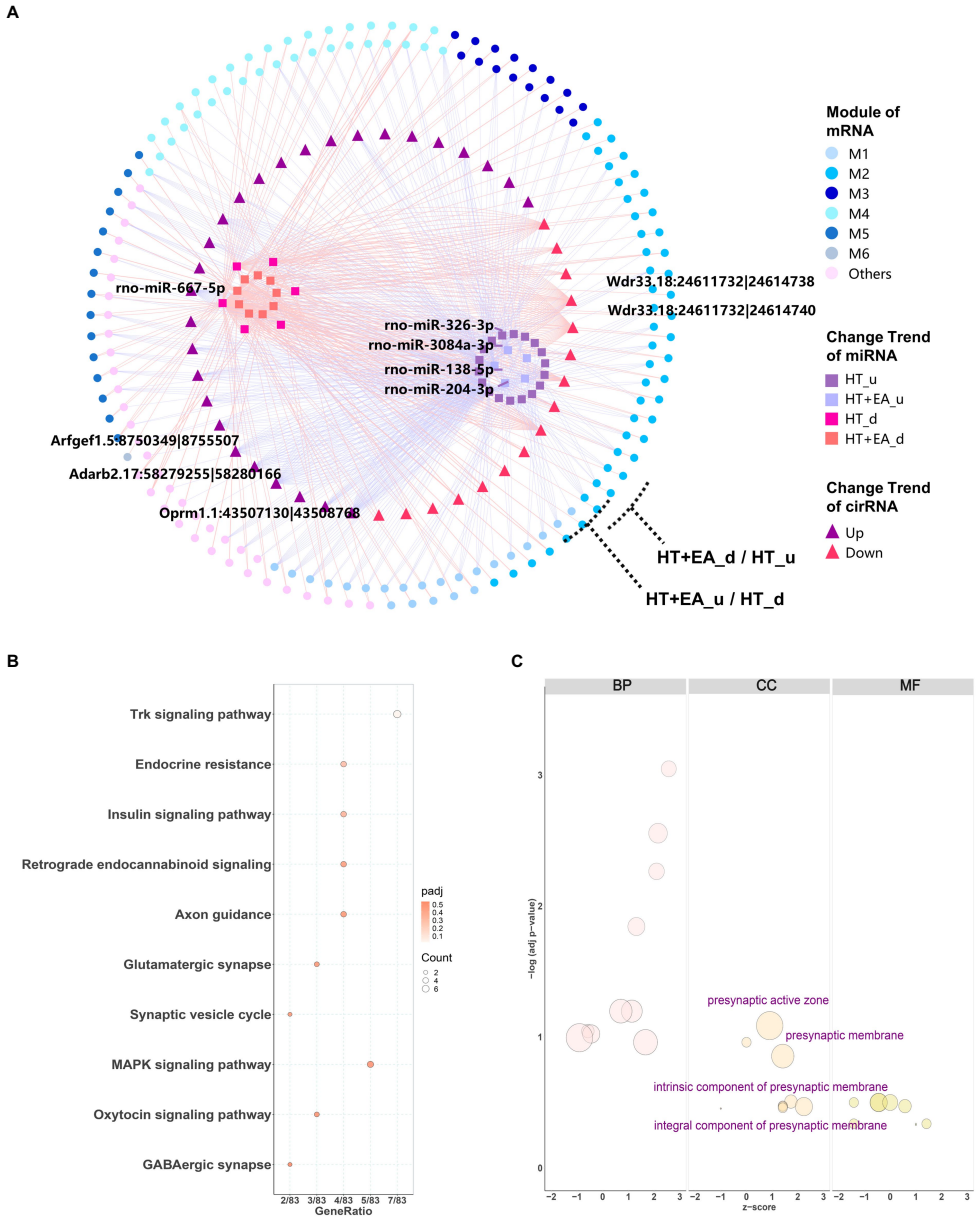
Interaction network and functional enrichment analysis of ncircRNA. **(A)** The circRNA-miRNA-mRNA interaction network showing circRNAs distributed among the six gene modules was composed of 44 circRNAs (squares), 38 miRNAs (triangles), and 399 mRNAs (circles). The top 5 weighted circRNAs and miRNAs were labelled based on their degree of connectiveness. **(B,C)** KEGG and GO functional enrichment analysis of circRNA-targeted mRNAs.

Considering the comprehensive relationship among circRNA, miRNA, and mRNA, the trends in changes in circRNA should be consistent with mRNA, while changes in miRNA should be the opposite of mRNA. Filtering by these rules removed 44 node circRNAs (ncircRNAs) out of 80 differentially expressed circRNAs backtracked from nmRNAs, and a total of 401 ncircRNA-nmiRNA-nmRNA networks were constructed (Figure 6A). Similar to the nmiRNA-mRNA network, the mRNAs with opposite change trends formed two outer rings, and nmiRNAs with the same change trends separately formed two inner clusters. Similar to the impact of nmiRNA regulation in different modules, M2 also had the largest proportion of genes regulated by ncircRNAs. Moreover, the top 2 weighted ncircRNAs, which were ADRB2 and OPRM1 regulators, were found to be related to M2, indicating that EA may exert its effect at least partly through circRNAs to regulate the stress and pain responses caused by surgical trauma. However, circRNA-regulated mRNAs only accounted for about 5% (117/2540) of the total DEmRNAs, indicating little effect of circRNAs in mediating the effects of EA treatment.

Functional enrichment analysis was also performed for the target genes regulated by ncircRNAs. KEGG analysis showed several more specific types of synapses, including endocannabinoid and glutamatergic synapses, as well as the Trk, insulin, and MAPK signaling pathways (Figure 6B). On the other hand, GO cellular component terms were shown to be related to presynapsis (Figure 6C), indicating that circRNAs regulate different aspects of cellular activities compared with miRNAs.

## 4. Discussion

**Figure 7:**
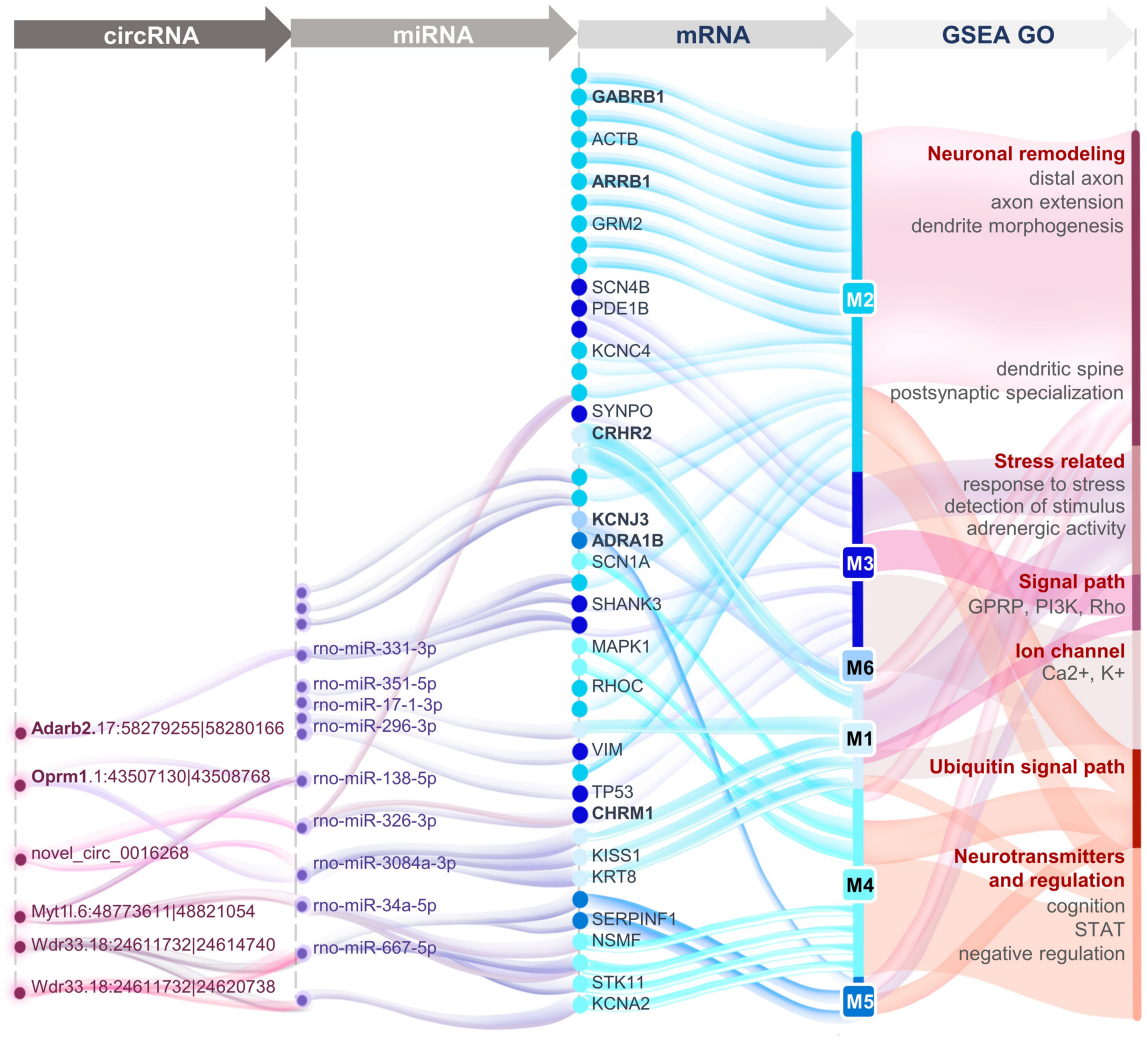
Panoramic view of the circRNA-miRNA-mRNA interaction networks in the PVN revealing the transcriptomic mechanisms of EA treatment for surgery-induced stress.

Dysfunction of the HPA axis has been suggested to be vital in the presence of surgery-induced stress, and this can be alleviated by EA treatment. The present study focused on the overall transcriptomic changes of two stress-related brain nuclei. Compared to the CEA, the transcriptional signature of the PVN was shown to be more closely associated with the effect of EA on the HPA axis, which is mainly responsible for neuron projection development and modulation of chemical synaptic transmission. In our HT rat model, network analysis revealed the possibility of EA acting through *OPRM1* and related circRNAs together with negatively regulating the sympathetic adrenergic system through *CRH* and *COMT*. Meanwhile, during EA treatment vascular contraction and dilation function mediated by *AGTR2* also synergized with neuroendocrine regulation, and negative regulation mainly functioned through GABAergic neurons. In addition, the small proportion of functional competing endogenous RNA indicated the reduced importance of indirect regulatory roles compared to direct transcription regulation (Figure 7). Sham EA remains controversial in different studies. Several parameters have been standardized for EA, including the location of acupoints, the depth of needle insertion, and the extent of electrical stimulation. We chose to remove the electrical stimulation for the SEA treatment and found little difference between SEA and HT groups, indicating the limited effects of SEA (Figures 2B,D,E). Considering the close relationship between the PVN, the CEA, and the neuroendocrine system, we selected these two brain regions for sequencing analysis. The results showed that PVN seems to be more relevant to EA function compared with the CEA. CEA function and its connectivity with the PVN remains to be further explored. Due to the limitation of transcriptome sequencing, the morphological changes and the single-cell resolution of gene regulation and neural connections were underestimated. The CEA can increase the activity of the PVN through direct projections (Davis and Shi, 1999), and neuron remodeling and epigenetic changes in the CEA can also exert effects on gene expression profiles in the PVN, especially under chronic stress conditions (McEwen et al., 2016).

Surgery plays increasingly important roles in modern clinical practice. However, surgery-induced stress encompasses a wide range of pathophysiological changes in the sympathetic nervous, endocrine, metabolic, immune, and hematopoietic systems (Desborough, 2000). Preoperative anxiety, surgical trauma, and postoperative pain inevitably cause hyperactivity of the HPA axis (Norman et al., 2008). Subsequent effects spread widely to the peripheral organs, such as cortisol surges from the adrenal gland, glucagon released from the pancreas, and thyroxine produced from the thyroid (Chernow et al., 1987). Thus, preoperative or postoperative stress interventions are needed that incorporate analgesics, psychological and rehabilitation treatments, etc. (El-Gabalawy et al., 2019). EA performed pre-, intra-, or post-operatively has been widely used as an analgesic (Pan et al., 2017; Qiao et al., 2020), and it is also beneficial for treating stress-induced endocrine disorders and neuropsychiatric disorders including epilepsy, neurosis, and diabetes (Yi et al., 2015; Liu et al., 2017; Protto et al., 2019). Previous studies reported that EA can reduce the levels of CRH and CORT in rats suffering from weightlessness-induced stress (Zhang et al., 2015). The mechanisms of EA involve specific acupoints that correspond to specific internal organs or brain regions. Based on this theory, EA at ST 36 has been shown to drive the vagal-adrenal anti-inflammatory axis (Liu et al., 2021). In our study, we constructed the HT rat model to simulate clinical surgical trauma and verified the hyperactivity of the HPA axis. EA was then performed at ST 36 and SP 6 to relieve the hyperactivity of the HPA axis, manifested as decreased expression of hypothalamic CRH and serum ACTH and CORT.

In order to further explore the targets of EA treatment, the gene expression profiles in the PVN and CEA were determined by RNAseq. GO and KEGG analysis revealed changes in neuroendocrine-related genes in the PVN and changes in ribosome-related genes in the CEA. PPI and hub gene analysis identified *CRH*-related subnetworks, indicating that the PVN is the brain region most strongly targeted by EA. In the PVN, CRH stimulates the HPA axis to respond to the stress together with the hypothalamus-pituitary-thyroid axis, in which it has been shown that CRH can act through CRH-R2 to regulate the secretion of thyrotropes (Groef et al., 2003). SynGAP is phosphorylated by CaMKII and is regulated by the stimulation of neurons with NMDA-type glutamate receptors, although another differentially regulation mediator – CDK5 – was not detected in our model (Walkup et al., 2015). GFAP, an astrocyte marker, appeared in the Hub gene network, also indicating that remodeling of astrocytes may function in the alleviation of stress by EA (Codeluppi et al., 2021). Stress-related genes were selected and used in the subsequent WGCNA and GSEA analysis. In addition to the matched genes, others such as *prostaglandin*, *E2*, *epinephrine*, *acetylcholine*, *IL1*, and *NF-κB* were also tested but without being matched. In the *CRH*-represented M1 module, *AVP* and *COMT* were found to be significantly co-expressed, which similarly has been shown by metabolomic analysis after single EA at ST 36 (Lee et al., 2020). The *MAPK1*-represented M4 module was functionally enriched with Wnt signaling and negatively regulated for signal transduction, among which *MAPK* family members, *GluN2A*, and cAMP-response element-binding protein (*CREB*) activity along with phosphorylation of ERK were shown to be suppressed by EA in our previous study (Wang et al., 2021).

Competing endogenous RNA also has been reported to regulate the neuroendocrine activity that is involved in stress responses (Nanayakkara et al., 2020), and several lines of evidence suggest that both miRNA and circRNA are involved in the mechanisms of EA. It has been reported that the expression of hypothalamic miRNA-142 is down-regulated after hepatectomy and that this can be reversed by EA treatment (Zhu et al., 2017). It has also been shown that co-transfection with miR-212 and CREB reduces the expression of cellular CRH, while the miR-212 antagomir significantly increases the level of CRH (Tang et al., 2017). Interestingly, the two most strongly EA-related miRNA-regulated genes, *MGLL* and *NSMF*, were filtered out. *MGLL* is localized mainly in the cannabinoid receptor type 1-enriched presynaptic region, and its inhibitor can potentially be used as a treatment for pain and stress dysfunctions (Abdel-Magid, 2022). *NSMF* in the PVN is mainly related to the migration of GnRH neurons, and it plays critical roles in the central regulation of reproduction (Louden et al., 2019).

CircRNA has been shown to participate in the process of neuroendocrine dysfunction, and it is a potential diagnostic markers for several disease like endocrine tumors (Zheng et al., 2020), cardiovascular diseases (Lu and Thum, 2019), and hypertension (Shen et al., 2021). EA is reported to ameliorate stress-related depression by regulating the circRNA-miRNA-mRNA networks involved in circadian rhythms and neurotransmitter transport (Zheng et al., 2019). We found adenosine deaminase and the endogenous opioid system to be strongly corelated with circRNA regulation after EA treatment. Up to now, the database of circRNA has been widely dispersed, and there have been only limited numbers of uniform and in-depth studies. In our experiment, two circRNAs, Adarb2.17:58279255|58280166 and Oprm1.1:43507130|43508768, were found to be the most relevant to EA treatment. The Adarb circRNA was predicted to function through rno-miR-204-3p to regulate *SYNGAP1* and *CHRM 1* and through rno-miR-331-3p to regulate *SHANK3*. The Oprm1 circRNA was predicted to negatively function on three different miRNAs, including rno-miR-34a-5p, rno-miR-138-5p, and rno-miR-3084-3p, and to regulate *KISS1*, *KRT8*, *STK11*, *RHOC*, and *TP53*. The circRNA of OPRM1 is highly conserved among humans and rodents and is primarily involved in the response to pain (Irie et al., 2019). Thus, the present study provides clues for miRNAs and circRNAs as potential therapeutic targets for neuroendocrine dysfunction, and these were also the underlying mechanisms of EA treatment. Further efforts are needed to develop a unified standard database for circRNAs.

This study had one limitation that should be mentioned. We only used a value of *p* threshold to filter out DEmRNAs without logFC, which otherwise could have resulted in the unexpected omission of a large proportion of important stress-related genes.

## 5. Conclusion

The transcriptomes of the PVN and CEA were determined in rat models of surgery-induced stress, and the PVN was found to be more related than the CEA to EA’s treatment effect on the central nervous system. Opioids and the adrenaline system play major roles in EA’s effects, partly regulated through their miRNAs and circRNAs. In addition, negative regulation of the vascular and GABAergic systems showed synergistic effects and thus showed the multiple targets of EA. Moreover, competing endogenous RNA appears to contribute in only a minor way to EA’s treatment effect on stress reduction. Thus, our study provides evidence for the overall gene profile expression changes and the competing endogenous RNA functions underlying the mechanisms of EA treatment.

## Data availability statement

The datasets presented in this study can be found in online repositories. The names of the repository/repositories and accession number(s) can be found in the article/Supplementary material.

## Ethics statement

The animal study was reviewed and approved by the Ethics Committee of Fudan University in China (20190221-068).

## Author contributions

YW, WH, and ZT conceived the experiments, designed the project and protocols, and developed the collaborations. JH and JZ performed the experiments. YW and WH analyzed the data and wrote the manuscript. YF, NJ, and ZT provided scientific oversight and guidance and edited the manuscript. YW, WH, YF, and ZT are the guarantors of this work and, as such, had full access to all of the data in the study and take responsibility for the integrity of the data and the accuracy of the data analysis. All authors contributed to the article and approved the submitted version.

## Funding

This work was supported by the National Natural Science Foundation of China (Grants numbers 81573712 and 81973639) and supported by the Innovative Research Team of High-level Local Universities in Shanghai. We appreciate the technical support provided by the Shanghai Key Laboratory for Acupuncture Mechanism and Acupoint Function (No. 21DZ2271800).

## Conflict of interest

The authors declare that the research was conducted in the absence of any commercial or financial relationships that could be construed as a potential conflict of interest.

## Publisher’s note

All claims expressed in this article are solely those of the authors and do not necessarily represent those of their affiliated organizations, or those of the publisher, the editors and the reviewers. Any product that may be evaluated in this article, or claim that may be made by its manufacturer, is not guaranteed or endorsed by the publisher.

## Figures and Tables

**Table 1:** 

EA	Electroacupuncture
HPA	Hypothalamic–pituitary–adrenal
HT	Hepatectomy
NC	Normal control
RNA-seq	RNA sequencing techniques
CRH	Corticotrophin-releasing hormone
ACTH	Adrenocorticotropic hormone
CORT	Corticosterone
PVN	Periventricular nucleus
CEA	Amygdala
GABA	Gamma-aminobutyric acid
NMDA	N-Methyl-D-aspartate
miRNAs	microRNAs
circRNAs	Circular RNAs
WGCNA	Weighted Gene Co-expression Network Analysis
GSEA	Gene Set Enrichment Analysis
PCA	Principle Component Analysis
KEGG	Kyoto Encyclopedia of Genes and Genomes
GO	Gene Ontology
PPI	Protein–protein interaction
nmiRNAs	Node miRNAs
*TRH*	Thyrotropin-releasing hormone
*Camk2a*	Calcium-dependent protein kinase II alpha
*SYNGAP1*	Synaptic Ras GTPase activating protein 1
*OPRM1*	Opioid receptor mu 1
*D2R*	Dopamine receptor D2
*MAPK1*	Mitogen-activated protein kinase 1
*AGTR2*	Angiotensin II receptor type 2
*GABRA1*	Gamma-aminobutyric acid type A receptor alpha1 subunit
*AVP*	Arginine vasopressin
*COMT*	Catechol-O-methyltransferase
*HTR1A*	Hydroxytryptamine receptor 1A
*GnRH*	Gonadotropin-releasing hormone
*MGLL*	Monoglyceride lipase
*NSMF*	Neuronal migration factor
ncircRNAs	Node circRNAs
*CREB*	cAMP-Response element-binding protein
