# Supplementary material for: Electroacupuncture alleviates perioperative hypothalamus-pituitary-adrenal axis dysfunction via circRNA-miRNA-mRNA networks
Source: Front Mol Neurosci. 2023 Jan 25;16:1115569. doi: 10.3389/fnmol.2023.1115569 (PMC9905746; doi:10.3389/fnmol.2023.1115569)
Supplement: Supplementary file 1 [file Data_Sheet_1.ZIP › Raw data/Fig1/Fig1C CRH protein/Entire Original Gels of CRH and β-tubulin.pdf]

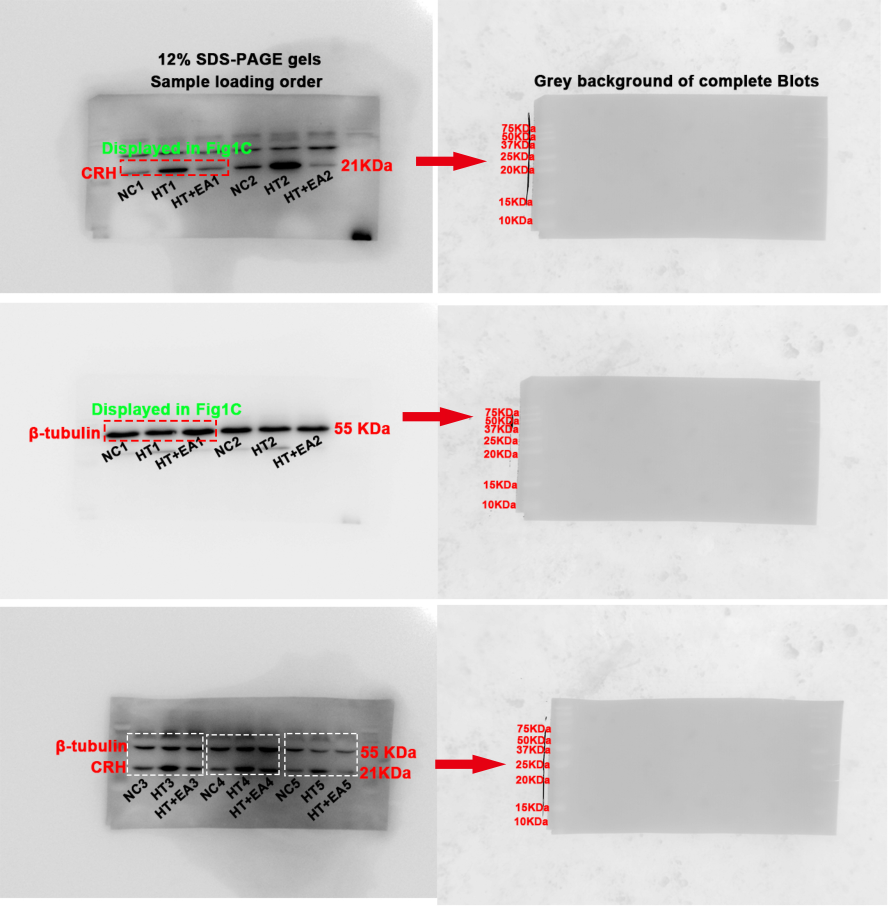
12% SDS-PAGE gels
Sample loading order
Displayed in Fig1C
CRH
21KDa
NC1
HT1
HT+EA1
NC2
HT2
HT+EA2
Grey background of complete Blots
75KDa
50KDa
37KDa
25KDa
20KDa
15KDa
10KDa
Displayed in Fig1C
β-tubulin
55 KDa
NC1
HT1
HT+EA1
NC2
HT2
HT+EA2
75KDa
50KDa
37KDa
25KDa
20KDa
15KDa
10KDa
β-tubulin
CRH
55 KDa
21KDa
NC3
HT3
HT+EA3
NC4
HT4
HT+EA4
NC5
HT5
HT+EA5
75KDa
50KDa
37KDa
25KDa
20KDa
15KDa
10KDa
