# Supplementary material for: Electroacupuncture alleviates perioperative hypothalamus-pituitary-adrenal axis dysfunction via circRNA-miRNA-mRNA networks
Source: Front Mol Neurosci. 2023 Jan 25;16:1115569. doi: 10.3389/fnmol.2023.1115569 (PMC9905746; doi:10.3389/fnmol.2023.1115569)
Supplement: Supplementary file 1 [file Data_Sheet_1.ZIP › Raw data/Fig3/Fig3C-F/Original_data_statement.docx]

Principle Component Analysis (PCA) and functional enrichment analysis, including KEGG and GO was produced by the integrated automatic analysis platform of Novo Gene Biotech Co. Ltd. (Beijing, China) based on R.
