# Supplementary material for: Electroacupuncture alleviates perioperative hypothalamus-pituitary-adrenal axis dysfunction via circRNA-miRNA-mRNA networks
Source: Front Mol Neurosci. 2023 Jan 25;16:1115569. doi: 10.3389/fnmol.2023.1115569 (PMC9905746; doi:10.3389/fnmol.2023.1115569)
Supplement: Supplementary file 1 [file Data_Sheet_1.ZIP › Raw data/Fig4/Fig4A-D/Statement.docx]

Protein-protein interaction (PPI) network was produced by using the online Search Tool for the Retrieval of Interacting Genes (STRING) database (<https://string-db.org/>).
