# Supplementary material for: Electroacupuncture alleviates perioperative hypothalamus-pituitary-adrenal axis dysfunction via circRNA-miRNA-mRNA networks
Source: Front Mol Neurosci. 2023 Jan 25;16:1115569. doi: 10.3389/fnmol.2023.1115569 (PMC9905746; doi:10.3389/fnmol.2023.1115569)

WGCNA was adapted from the open source (<https://bmcbioinformatics.biomedcentral.com/articles/10.1186/1471-2105-9-559>, <https://horvath.genetics.ucla.edu/html/CoexpressionNetwork/Rpackages/WGCNA/> ), and GSEA was produced by the GSEA software (<https://www.gsea-msigdb.org/gsea/index.jsp> ).

All visualization was generated by the Cytoscape (free access, <https://cytoscape.org/> ) and Graphpad Prisma (GraphPad Software Inc., San Diego, CA, USA).


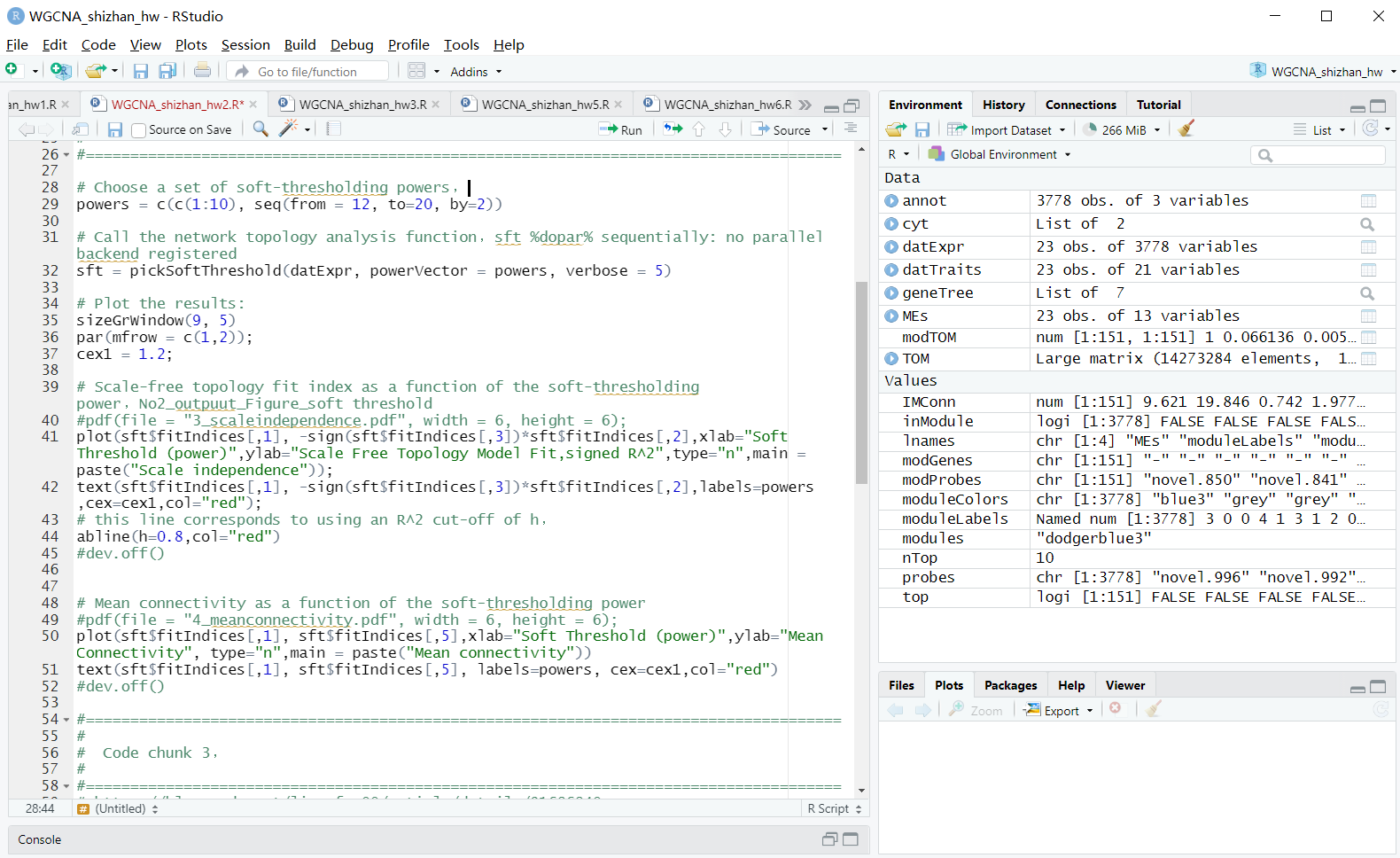

Supplement: Supplementary file 1 [file Data_Sheet_1.ZIP › Raw data/Fig4/Fig4E/Statement.docx]
