# Supplementary figures and images for: Electroacupuncture alleviates perioperative hypothalamus-pituitary-adrenal axis dysfunction via circRNA-miRNA-mRNA networks
Source: Front Mol Neurosci. 2023 Jan 25;16:1115569. doi: 10.3389/fnmol.2023.1115569 (PMC9905746; doi:10.3389/fnmol.2023.1115569)

# CEAEAvsCEAHT

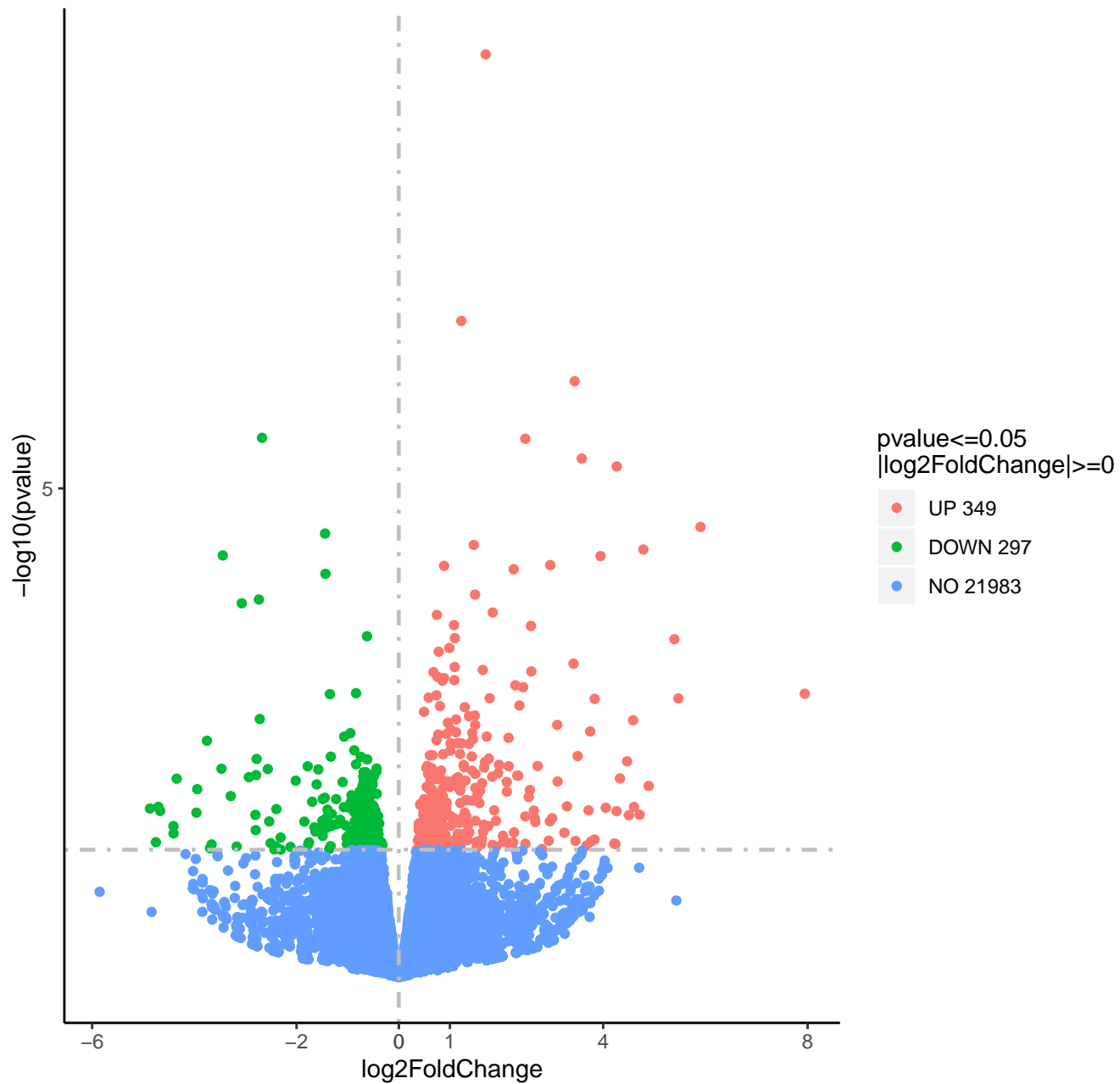

Supplement: Supplementary file 1 [file Data_Sheet_1.ZIP › Raw data/Fig2/Fig2C/CEA/EA VS HT/CEAEAvsCEAHT_volcano.pdf]

# CEAHTvsCEANC

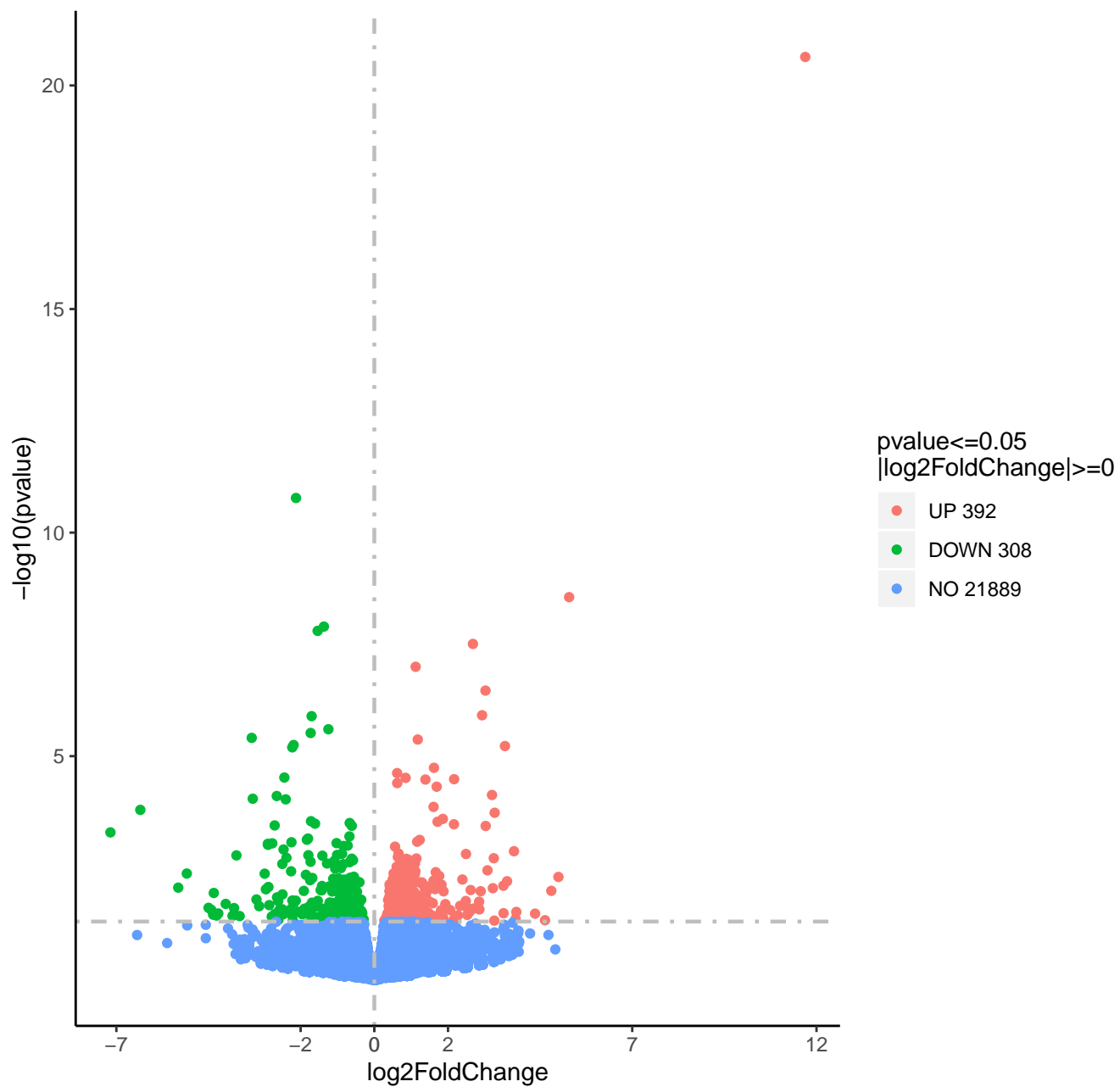

Supplement: Supplementary file 1 [file Data_Sheet_1.ZIP › Raw data/Fig2/Fig2C/CEA/HT VS NC/CEAHTvsCEANC_volcano.pdf]

## EAvsHT

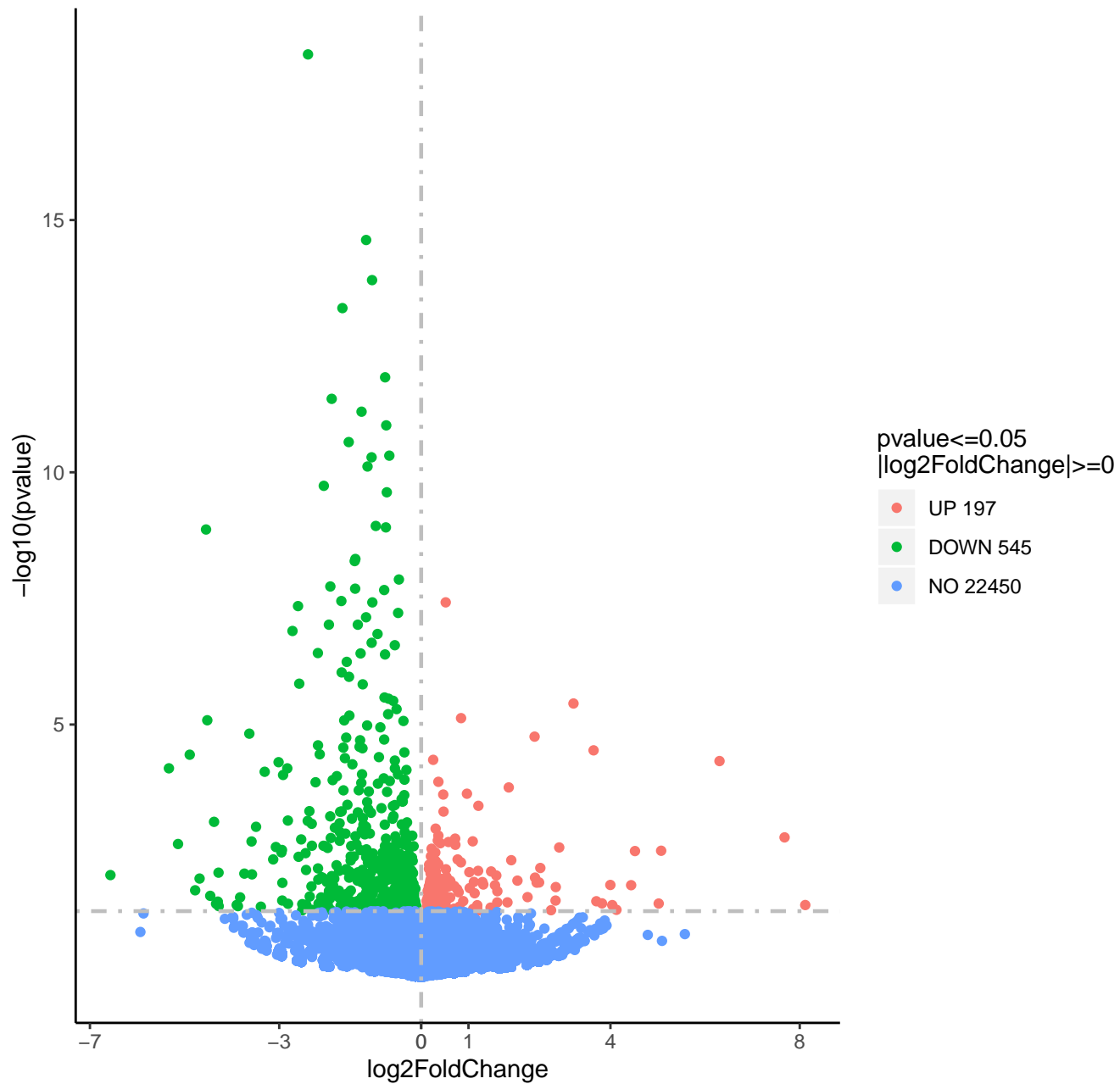

Supplement: Supplementary file 1 [file Data_Sheet_1.ZIP › Raw data/Fig2/Fig2C/PVN/EA VS HT/EAvsHT_volcano.pdf]

# HTvsNC

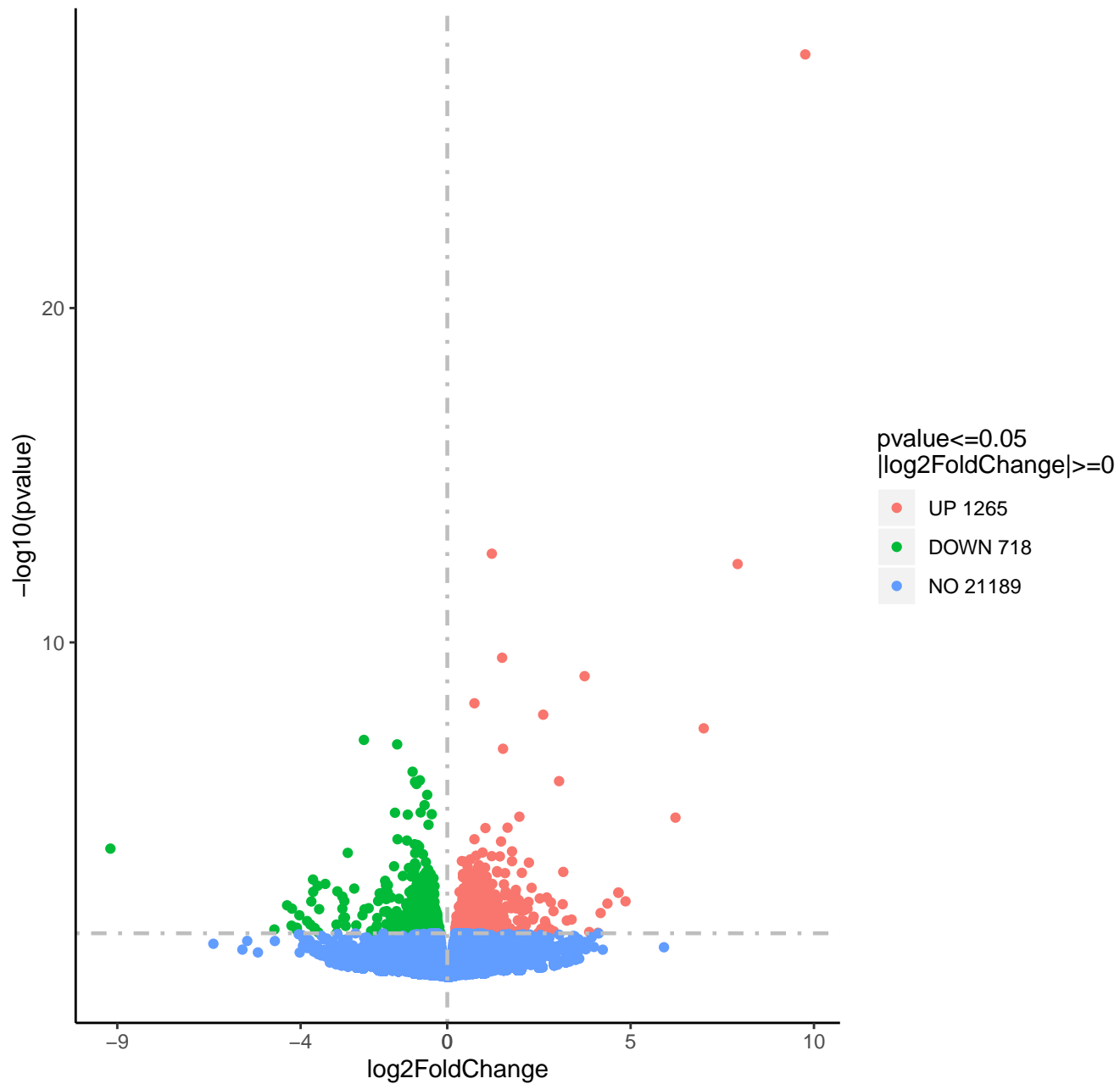

Supplement: Supplementary file 1 [file Data_Sheet_1.ZIP › Raw data/Fig2/Fig2C/PVN/HT VS NC/HTvsNC_volcano.pdf]

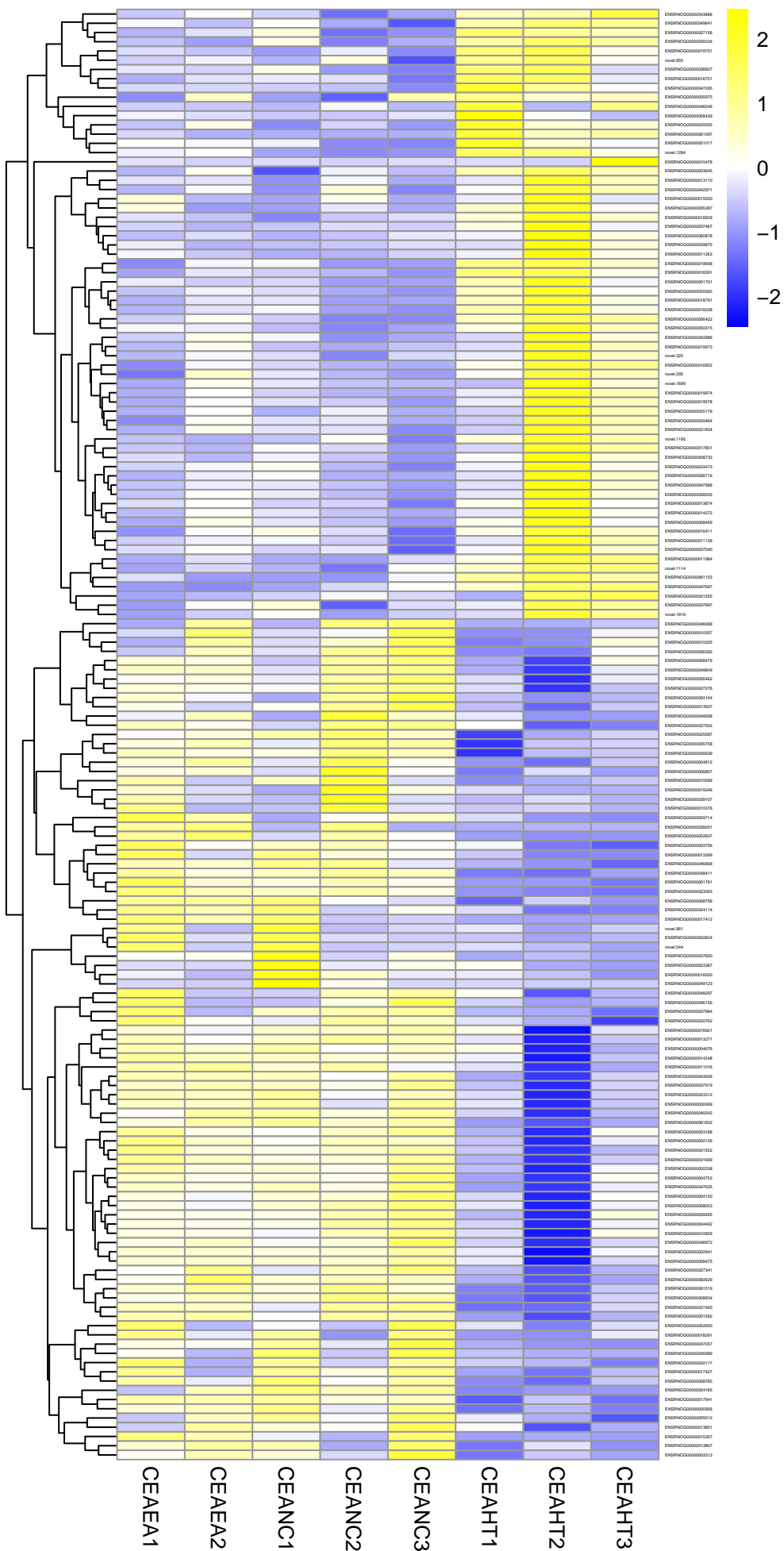

Supplement: Supplementary file 1 [file Data_Sheet_1.ZIP › Raw data/Fig2/Fig2D/heatCluster_CEADEmRNACluster.detail.pdf]

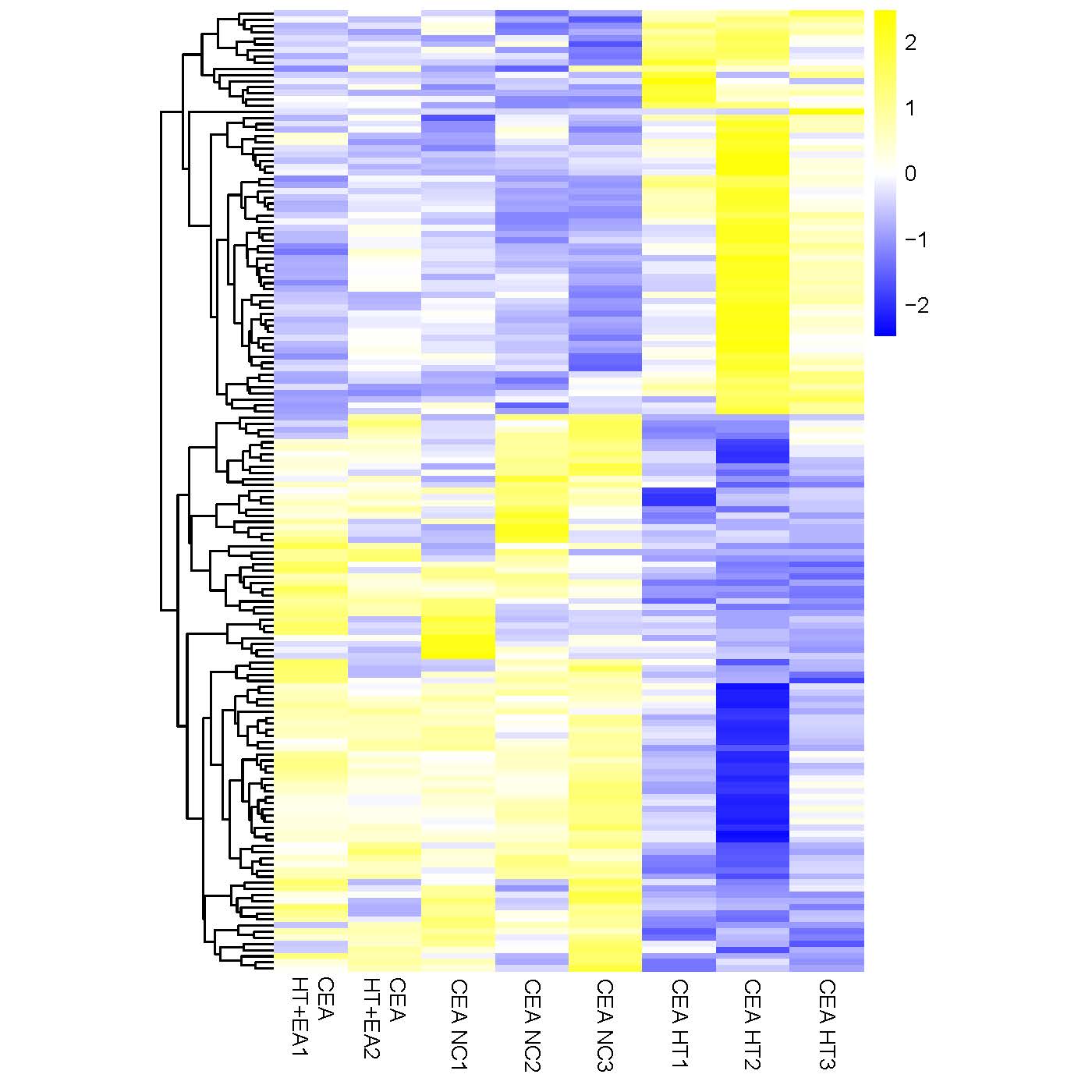

Supplement: Supplementary file 1 [file Data_Sheet_1.ZIP › Raw data/Fig2/Fig2D/heatCluster_CEADEmRNACluster.jpg]

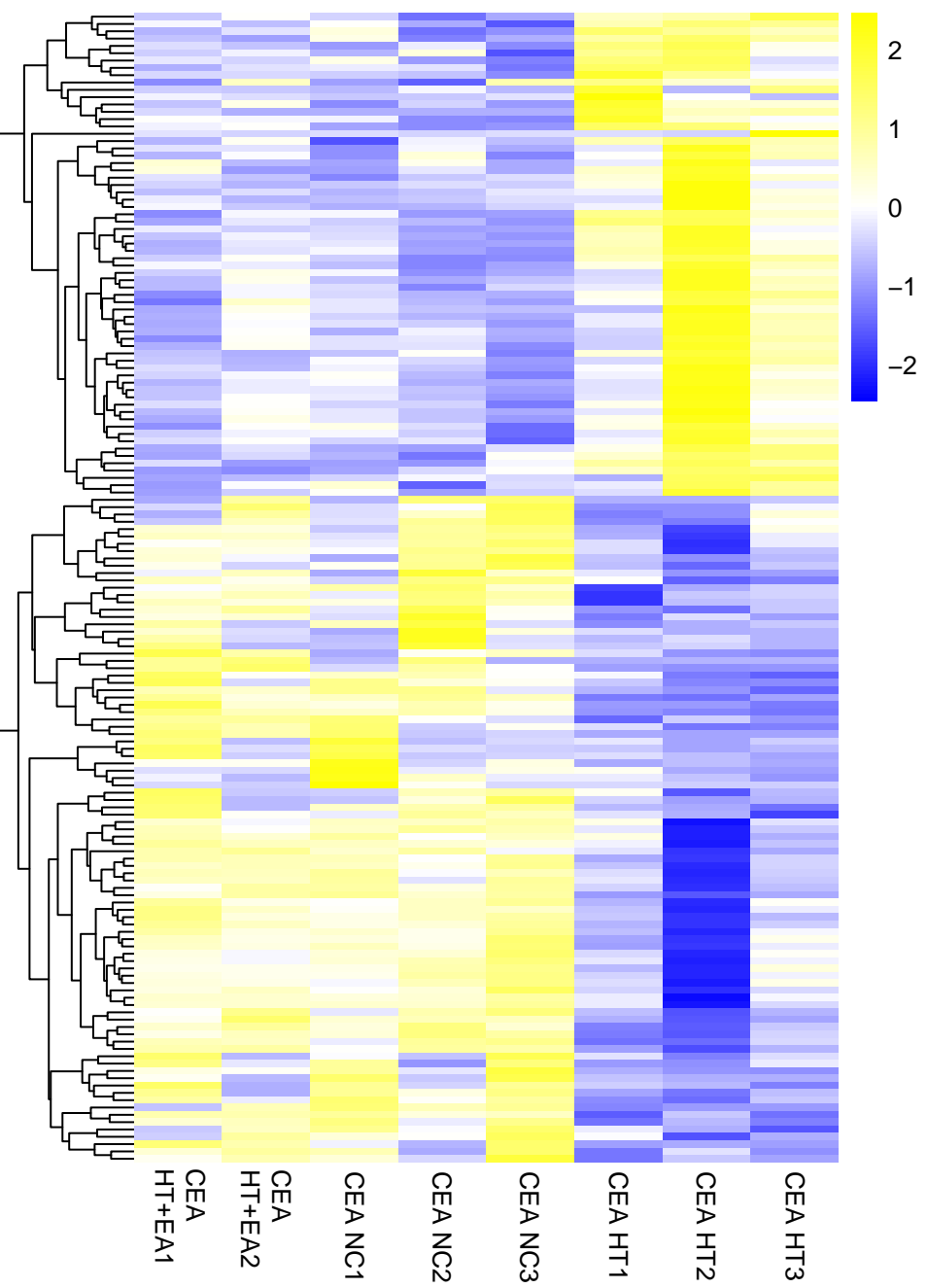

Supplement: Supplementary file 1 [file Data_Sheet_1.ZIP › Raw data/Fig2/Fig2D/heatCluster_CEADEmRNACluster.pdf]

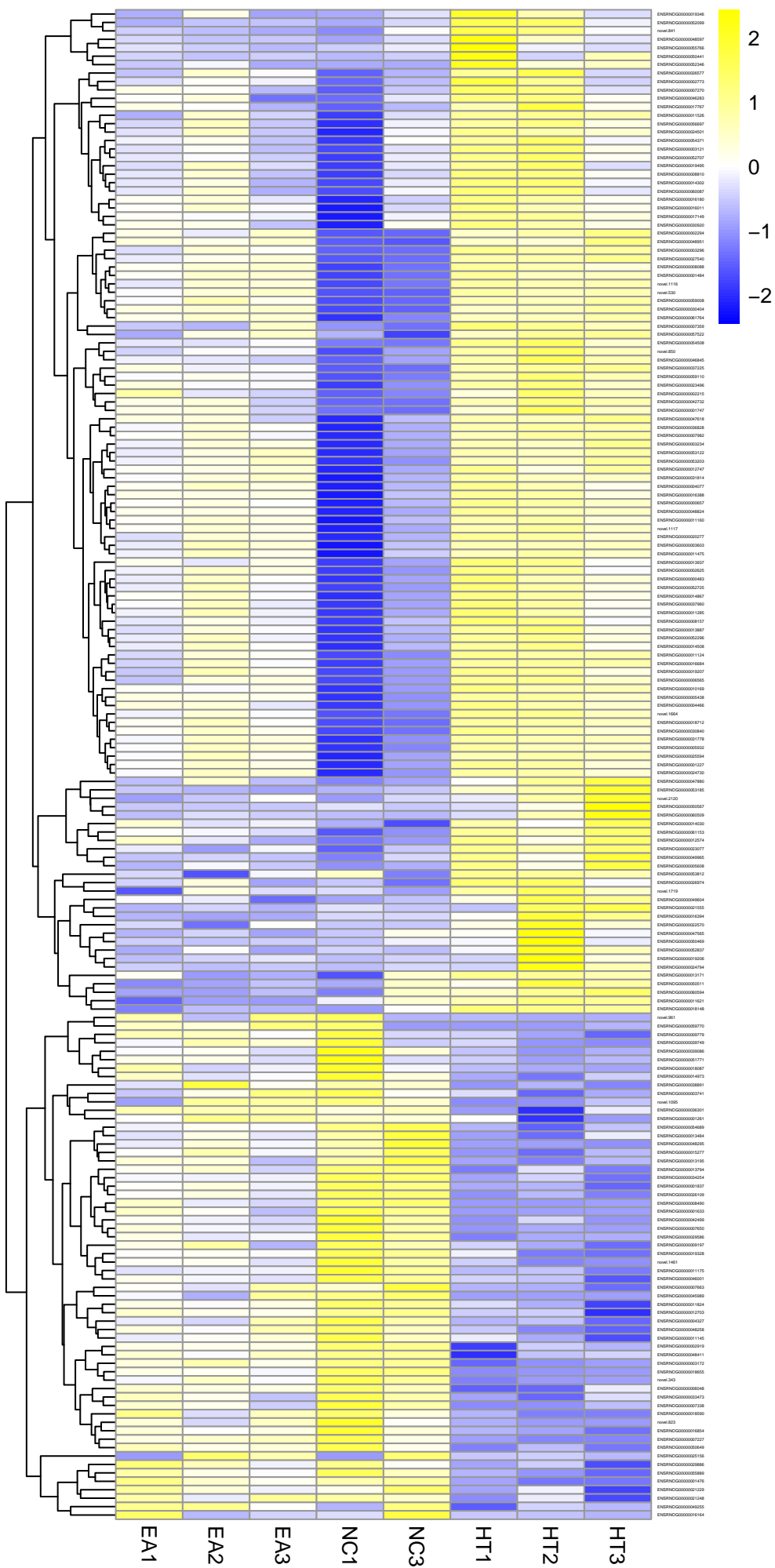

Supplement: Supplementary file 1 [file Data_Sheet_1.ZIP › Raw data/Fig2/Fig2E/heatCluster_PVNDEmRNACluster.detail.pdf]

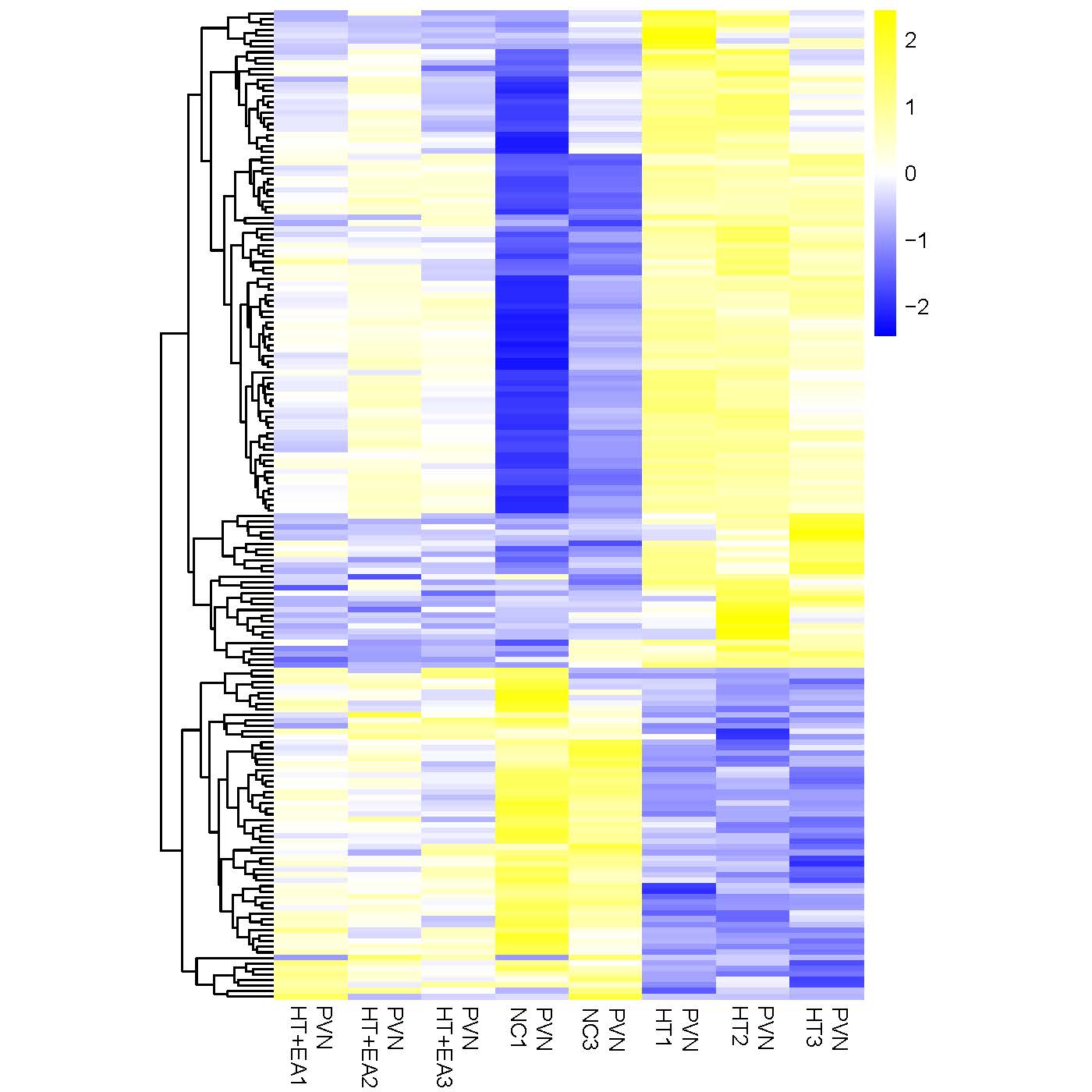

Supplement: Supplementary file 1 [file Data_Sheet_1.ZIP › Raw data/Fig2/Fig2E/heatCluster_PVNDEmRNACluster.jpg]

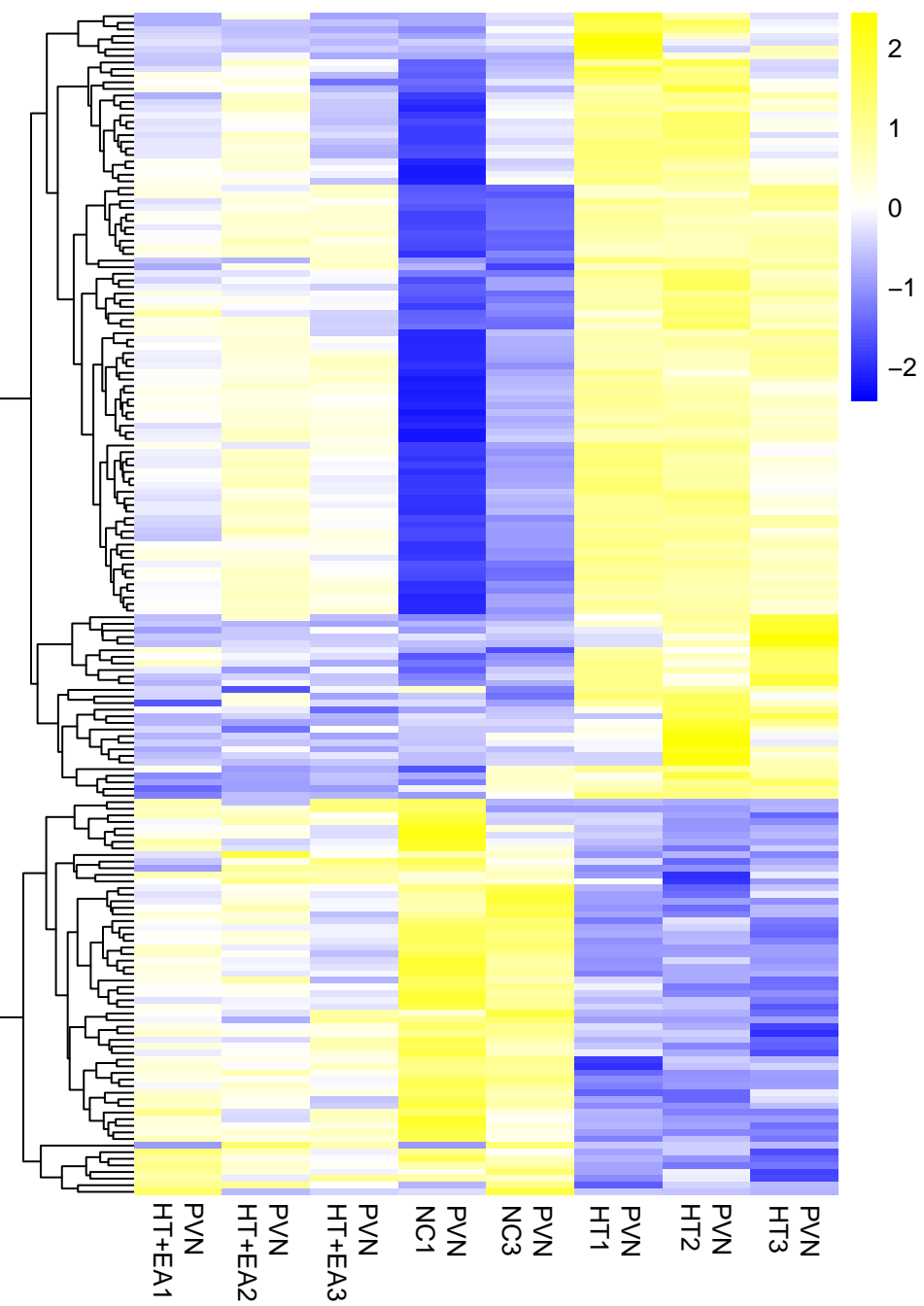

Supplement: Supplementary file 1 [file Data_Sheet_1.ZIP › Raw data/Fig2/Fig2E/heatCluster_PVNDEmRNACluster.pdf]
