# Supplementary material for: Electroacupuncture alleviates perioperative hypothalamus-pituitary-adrenal axis dysfunction via circRNA-miRNA-mRNA networks
Source: Front Mol Neurosci. 2023 Jan 25;16:1115569. doi: 10.3389/fnmol.2023.1115569 (PMC9905746; doi:10.3389/fnmol.2023.1115569)
Supplement: Supplementary file 2 [file Data_Sheet_2.ZIP › Supplementary files/Supplementary table.docx]

**Supplementary Table 1. Primers and sequences**

| **Primer** | **Sequence (5' to 3')** |
| --- | --- |
| CRH F | CTCTCTGGATCTCACCTTCCAC |
| CRH R | CTAAATGCAGAATCGTTTTGGC |
| GAPDH F | GTATGACTCTACCCACGGCAAGT |
| GAPDH R | TTCCCGTTGATGACCAGCTT |

**Supplementary Sheet 1:** A total of 1246 and 2725 DEmRNAs were identified in the CEA and PVN.

**Supplementary Sheet 2:** GO and KEGG analysis of nmRNA in the CEA and PVN.

**Supplementary Sheet 3:** Data flow of the predicted miRNAs and nmRNAs, predicted target mRNAs, and DEmRNAs.
